# *Burkholderia pseudomallei* produces 2-alkylquinolone derivatives important for host virulence and competition with bacteria that employ naphthoquinones for aerobic respiration

**DOI:** 10.3389/fmicb.2024.1474033

**Published:** 2024-10-14

**Authors:** Sherry Mou, Viktoriia Savchenko, Verena Filz, Thomas Böttcher, David DeShazer

**Affiliations:** ^1^Foundational Sciences Directorate, Bacteriology Division, United States Army Medical Research Institute of Infectious Diseases, Frederick, MD, United States; ^2^Faculty of Chemistry and Department of Microbiology and Ecosystem Science, University of Vienna, Vienna, Austria

**Keywords:** pathogenesis, select agent, natural products, biosynthetic gene cluster, isoprenoid quinone

## Abstract

Melioidosis is caused by *Burkholderia pseudomallei*, an opportunistic Gram-negative pathogen that inhabits soil and water in tropical and subtropical regions. *B. pseudomallei* infections often occur following contact with contaminated water or soil or by inhalation of contaminated dust and water droplets. There is limited knowledge about how *B. pseudomallei* is able to survive in harsh environmental conditions and compete with the microbes that inhabit these niches. Previous research demonstrated that 3-methyl-2-alkylquinolones (MAQs), and their corresponding *N*-oxides (MAQNOs), are produced by *B. pseudomallei* and provide a competitive advantage when grown in the presence of Gram-positive bacteria. In this study, 39 Gram-negative environmental bacteria in the *Pseudomonadota* and *Bacteroidota* phyla were isolated and characterized. Intriguingly, *B. pseudomallei* inhibited 71% of bacteria in the phylum *Bacteroidota* in zone of inhibition and coculture competition assays, but no *Pseudomonadota* isolates were similarly inhibited. Transposon mutagenesis was utilized to identify *B. pseudomallei* genes required for the inhibition of *Sphingobacterium* sp. ST4, a representative member of the *Bacteroidota*. Three mutations mapped to *hmqA-G*, the locus encoding 2-alkylquinolone derivatives, and two mutations were identified in *scmR*, a gene encoding a quorum-sensing controlled LysR-type transcriptional regulator. *B. pseudomallei* strains with deletion mutations in *hmqD* and *scmR* were unable to produce 2-alkylquinolone derivatives or inhibit *Bacteroidota* isolates in competition assays. RAW264.7 murine macrophage cells were infected with *B. pseudomallei* 1026b and 1026b *ΔhmqD* and there was a 94-fold reduction in the number of intracellular 1026b *ΔhmqD* bacteria relative to 1026b. The 50% lethal dose (LD_50_) of 1026b and 1026b *ΔhmqD* in BALB/c mice was determined to be 3 x 10^5^ colony forming units (CFU) and > 1 x 10^6^ CFU, respectively. Taken together, the results indicate that the products of the *B. pseudomallei hmqA-G* locus are important for intracellular replication in murine macrophages, virulence in a mouse model of melioidosis, and competition with bacteria that utilize naphthoquinones for aerobic respiration.

## Introduction

*Burkholderia pseudomallei*, the etiologic agent of melioidosis, is a Gram-negative bacterium found in water and soil in tropical and subtropical environments worldwide (Inglis and Mayo, 2012; Limmathurotsakul et al., 2016; Limmathurotsakul et al., 2012; Meumann et al., 2024). Humans and animals are exposed to the pathogen via inhalation, ingestion, and/or direct contact with contaminated water or soil. Melioidosis patients often present with bacteremia and/or pneumonia, but infections can also affect multiple organs and lead to a variety of clinical manifestations. People with diabetes, liver disease, renal disease, chronic lung disease, and other immunocompromising conditions are more likely to develop melioidosis than individuals without known risk factors. The global distribution of *B. pseudomallei* continues to grow (Currie et al., 2023) and a recent report demonstrates that it’s now endemic in the Mississippi Gulf Coast region of the United States (Petras et al., 2023). *B. pseudomallei* is classified as a Tier 1 Select Agent because it is infectious by the aerosol route, it is relatively difficult to treat with antibiotics, and no approved vaccine is currently available (Meumann et al., 2024).

Members of the genus *Burkholderia* harbor a diverse array of biosynthetic gene clusters (BGCs) that encode natural products with potential uses in medicine and agriculture (Klaus et al., 2020a; Kunakom and Eustáquio, 2019). As many as 21 distinct BGCs have been bioinformatically identified in the genomic sequences of *B. pseudomallei* strains and a majority of the encoded bioactive compounds have been chemically and biologically characterized (Biggins et al., 2014; Klaus et al., 2020a; Kunakom and Eustáquio, 2019). *B. pseudomallei* and *B. thailandensis*, a closely-related nonpathogenic species, both produce a variety of 2-alkylquinolone derivatives, including 2-alkylquinolones (AQs), AQ *N*-oxides (AQNOs), 3-methyl-2-alkylquinolones (MAQs), and MAQ *N*-oxides (MAQNOs) (Diggle et al., 2006; Klaus et al., 2020a; Klaus et al., 2020b; Mou et al., 2021; Piochon et al., 2020; Szamosvári et al., 2020; Vial et al., 2008). In fact, the biosynthetic enzymes encoded by the *hmqA-G* operon, and the unlinked *hmqL* gene, produce greater than twenty 2-alkylquinolone derivatives with unsaturated or saturated alkyl groups of seven to eleven carbons at the 2-position, a proton or methyl group at the 3-position, and molecules with a *N*-oxide at the 1-position (Diggle et al., 2006; Okada et al., 2016; Saalim et al., 2020; Savchenko et al., 2024; Vial et al., 2008; Wu and Seyedsayamdost, 2017). The variety of chemical substitutions on the AQ core can result in important differences in biological activity, which can include antibacterial, antimalarial, antifungal, antialgal, and antioxidant properties (Dow et al., 2020; Piochon et al., 2020; Saalim et al., 2020; Szamosvári et al., 2019).

In order to survive and persist in environmental niches, bacteria must compete with complex mixtures of microorganisms for limited nutrients (Srinivasan et al., 2024). Previous studies have demonstrated that *B. pseudomallei* and *B. thailandensis* effectively compete with environmental Gram-positive bacteria in a *hmqA-G*-dependent manner (Klaus et al., 2020b; Mou et al., 2021). In addition, Gram-positive bacteria are more effectively inhibited by chemically synthesized AQs, AQNOs, MAQs, and MAQNOs than Gram-negative bacteria (Piochon et al., 2020; Szamosvári and Böttcher, 2017; Szamosvári et al., 2020). The diverse 2-alkylquinolone compounds produced by the *Burkholderia hmq* genes may act synergistically to inhibit bacterial growth by acting on different targets (Klaus et al., 2020b; Wu and Seyedsayamdost, 2017). Wu and Seyedsayamdost demonstrated that 4-hydroxy-3-methyl-2-(2-nonenyl) quinoline (HMNQ) and 2-heptyl-4(1*H*)-quinoline *N*-oxide (HQNO) inhibit pyrimidine biosynthesis by acting on a common target, but interrupt the proton motive force by acting on different targets (Wu and Seyedsayamdost, 2017). It has been proposed that MAQNOs mimic the native bacterial menaquinone (MK)/menaquinol (MKH_2_) redox couple that shuttle electrons in the respiratory chain (Nowicka and Kruk, 2010; Szamosvári and Böttcher, 2017). MK is the most ancient type of isoprenoid quinone and is the only quinone used by Gram-positive bacteria for aerobic respiration (Franza and Gaudu, 2022; Schoepp-Cothenet et al., 2013). By comparison, the model Gram-negative bacterium *Escherichia coli* produces MK, demethylmenaquinone (DMK), and ubiquinone (UQ), but only employs UQ for aerobic respiration.

The goal of this study was to isolate a diverse collection of Gram-negative environmental bacteria and evaluate their ability to compete with *B. pseudomallei* in zone of inhibition and coculture assays. The results demonstrated that ∼13% of the environmental isolates were unable to survive and grow as well in coculture with *B. pseudomallei* as they did in monoculture. Further evaluation of the molecular mechanism by which *B. pseudomallei* inhibited the susceptible bacteria indicated that the inhibition was dependent on the production of AQ derivatives encoded by the *hmqA-G* gene cluster. Transcriptional profiling also indicated that the expression of the *hmqA-G* operon is dependent on ScmR, a LysR-type transcriptional regulator (LTTR) that is positively regulated by quorum sensing (QS). Infection of RAW 264.7 murine macrophage cells with *B. pseudomallei* 1026b and 1026b *ΔhmqD* revealed an intracellular replication defect in the mutant. The relative virulence of these strains was also assessed in BALB/c mice and 1026b *ΔhmqD* was found to be less virulent than 1026b in this animal model of melioidosis. Furthermore, a previously described *Burkholderia ambifaria* HmqD enzyme inhibitor exhibited strong activity against the production of MAQs and MAQNOs by *B. pseudomallei* and may represent a novel melioidosis therapeutic countermeasure. The results show that the wide variety of AQ, AQNO, MAQ, and MAQNO molecules produced by *B. pseudomallei* are important for virulence in a mammalian model of infection and are critical for competition with Gram-negative bacteria that utilize naphthoquinones such as MK and DMK for aerobic respiration.

## Materials and methods

### Bacterial strains, plasmids, and growth conditions

The bacterial strains and plasmids used in this study are shown in Supplementary Table 1. *Escherichia coli* and *B. pseudomallei* were grown at room temperature (RT) or 37°C on Luria-Bertani (LB) agar (Lennox formulation; Sigma-Aldrich) or in LB broth. One hundred micrograms per milliliter adenine HCl and 5 μg/ml thiamine HCl were added to solid and liquid media for growth of the *purM* select agent exempt strain *B. pseudomallei* Bp82 and mutant derivatives. Broth cultures were grown in 14-ml Falcon round-bottom polypropylene tubes with snap caps (Fisher Scientific) or in 13 ml polypropylene tubes with snap caps (Sarstedt) containing 3 ml of LB and shaken at 250 rpm unless indicated otherwise. When appropriate, antibiotics were added at the following concentrations: 25 μg/ml kanamycin (Km) and streptomycin (Sm) for *E. coli*, and 25 μg/ml polymyxin B (Pm) and 500 to 1,000 μg/ml Km for *B. pseudomallei*. Growth curves for Bp82 and CM139 were conducted by growing the bacteria in LB broth for 18 h at 37°C and 180 rpm. One hundred fifty microliters of saturated culture were inoculated into 10 ml of LB medium in a sterile 50 ml polypropylene tube with a screw cap (VWR). The caps of the tubes were loosely opened by a 180-degree turn and fixed in this position with tape to ensure equal oxygen supply. The cultures were incubated at 37°C and 180 rpm and growth evaluated at 1.5, 3.0, 4.5, 6.0, 7.5, 9.0, and 24 h by measurement of the OD_600_ of 250 μl of bacterial culture in plastic cuvettes. The experiment was performed in biological triplicates. A 20 μg/ml stock solution of the chromogenic indicator 5-bromo-4-chloro-3-indolyl-β-D-galactopyranoside (X-Gal) was prepared in *N*,*N*-dimethylformamide, and 40 μl was spread onto the surface of plate medium for blue/white screening in *E. coli* TOP10 or *E. cloni*^®^ 10G chemically competent cells.

Environmental bacteria were isolated from several sources in Frederick, MD, USA (latitude and longitude coordinates, 39.396509 and 277.368223, respectively) from 2017 to 2022. Bacterial river (R) isolates were obtained by spreading aliquots of Monocacy River water serially diluted in sterile phosphate-buffered saline (PBS) onto the surfaces of sheep blood agar plates, LB agar plates, LB agar plates supplemented with 60 μg/ml X-Gal, BD Difco M9 Minimal Salts (Becton, Dickinson and Company) agar plates containing 0.4% glucose, and Difco™ R2A agar (BD) plates. The plates were incubated at 37°C for 2 days, and colonies that could be easily distinguished from *B. pseudomallei* colonies on solid medium due to morphology, pigmentation, and/or β-galactosidase production were selected for further characterization. Bacterial colonies that could not easily be differentiated from *B. pseudomallei* colonies were also retained for further analysis. A similar strategy was employed for water obtained from a stream (ST) that flows directly into the Monocacy River. River sediment (RS) was obtained using sterile 50 ml conical tubes, resuspended in PBS, serially diluted in PBS, and spread onto agar plates as described above. Bacterial rhizosphere (RZ) isolates associated with the root microbiome of lawn weeds were placed into sterile 50 ml conical tubes and resuspended in PBS, vigorously vortexed, and serially diluted in PBS, and aliquots were spread onto agar plates.

Unique bacterial colonies were selected and PCR products were generated from purified genomic DNA using the universal 16S rRNA primers 533F (Weisburg et al., 1991) and 1492R (Lane, 1991). The nucleotide sequences of the partial 16S rRNA genes were used to search against the nonredundant nucleotide collection database using BLASTN (McGinnis and Madden, 2004) and the top nucleotide hits indicated that 39 distinct Gram-negative bacteria were isolated.

### DNA manipulation

Restriction enzymes (Roche Molecular Biochemicals and New England BioLabs), Antarctic phosphatase (New England BioLabs), and T4 DNA ligase (Roche Molecular Biochemicals) were used according to the manufacturer’s instructions. When necessary, the End-It DNA End-Repair kit (Epicentre) was used to convert 5′ or 3′ protruding ends to blunt-ended DNA. The DNA fragments used in the cloning procedures were excised from agarose gels and purified with a PureLink Quick Gel Extraction Kit (Thermo Fisher Scientific). Bacterial genomic DNA was prepared from overnight LB broth cultures with the GenElute Bacterial Genomic DNA Kit (Sigma-Aldrich). Plasmids were purified from overnight LB broth cultures by using the Wizard Plus SV Miniprep DNA Purification System (Promega).

### PCR amplifications

The PCR primers used in this study are shown in Supplementary Table 1. The PCR products were sized and isolated by using agarose gel electrophoresis, cloned using the pCR2.1-TOPO TA Cloning Kit (Life Technologies), and transformed into chemically competent TOP10 or *E. cloni*^®^ 10G. The PCR amplifications were performed in a final reaction volume of 50 or 100 μl containing 1x FailSafe PCR PreMix D (Epicentre), 1.25 U FailSafe PCR Enzyme Mix (Epicentre), 1 μM PCR primers, and approximately 200 ng of genomic DNA. Genomic DNA was isolated from all environmental bacterial isolates, and their 16S rRNA genes were PCR amplified using the primers 533F and 1492R (Supplementary Table 1) and cloned into pCR2.1-TOPO. Colony PCR was utilized to screen for *B. pseudomallei* deletion mutants. Briefly, sucrose-resistant and Km-sensitive colonies were resuspended in 50 μl of water, and 2 μl were added to the PCR mixture rather than purified genomic DNA. PCR cycling was performed using a Mastercycler pro S (Eppendorf) and heated to 97°C for 5 min. This was followed by 30 cycles of a three-temperature cycling protocol (97°C for 30 s, 55°C for 30 s, and 72°C for 1 min) and 1 cycle at 72°C for 10 min. The scmR-5′-F/scmR-5′-R and scmR-3′-F/scmR-3′-R PCR products were joined by splicing by overlap extension (SOE) and the resulting product was incorporated into pMo130 using the In-Fusion HD EcoDry Cloning Kit (Takara Bio USA, Inc) to construct Bp82 *ΔscmR* (Supplementary Table 1). For PCR products larger than 1 kb, an additional 1 min per kb was added to the extension time.

### DNA sequencing

DNA inserts cloned into pCR2.1-TOPO were PCR amplified with M13 forward and M13 reverse primers (Supplementary Table 1), and unincorporated deoxynucleoside triphosphates (dNTPs) and primers were removed using the DyeEx 2.0 Spin Kit (Qiagen). The PCR products were then sequenced with the M13 forward and M13 reverse primers using the ABI BigDye Terminator v3.1 Cycle Sequencing Kit (Thermo Fisher Scientific) and an Applied Biosystems SeqStudio Genetic Analyzer (Thermo Fisher Scientific) according to the manufacturer’s instructions. The nucleotide sequences were analyzed with DNASTAR Lasergene 17 software.

### Tn*Mod*-OKm’ mutagenesis and plasmid conjugations

Tn*Mod*-OKm’ (Dennis and Zylstra, 1998) was delivered to Bp82 via conjugation with *E. coli* S17-1 (pTn*Mod*-OKm’) by using a membrane filter mating technique. Briefly, S17-1 (pTn*Mod*-OKm’) was inoculated into 3 ml of LB broth containing Km and Sm and grown at 37°C for 18 to 20 h with shaking (250 rpm). *B. pseudomallei* was also grown under these conditions but without antibiotic selection. One hundred microliters of each saturated culture were added to 3ml of sterile 10 mM MgSO_4_, mixed, and filtered through a 0.45-mm-pore-size nitrocellulose filter using a 25-mm Swinnex filter apparatus (Millipore). Filters were placed on LB plates supplemented with 10 mM MgSO_4_ and incubated for 8 h in a 37°C incubator. The filters were washed with 1-2 ml of sterile phosphate-buffered saline (PBS), and 100–300 μl aliquots were spread onto LB agar plates containing Km and Pm. Km^r^ and Pm^r^ colonies were identified after 48 h of incubation at 37°C. Tn*Mod*-OKm’ contains a Km^r^ gene and a pMB1 conditional origin of replication that does not function in *B. pseudomallei*, allowing the rapid cloning of DNA adjacent to the transposon’s site of insertion into *E. coli*. The in vitro cloning of DNA flanking the Tn*Mod*-OKm’ insertion sites in *B. pseudomallei* CCM1, CCM2, CCM6, CCM7, and CCM9 was performed by digesting total genomic DNA with the restriction endonucleases *Asc*I, *Sal*I or *Not*I, self-ligating, and transforming into an *E. coli* host (Supplementary Table 1). The resulting plasmids were then sequenced with outward facing primers (TnMod-LT2 or KM-RT) that binds to the left or right end of Tn*Mod*-OKm’. The resulting sequence reactions revealed the junction of the transposon and *B. pseudomallei* genomic DNA. Plasmids pMo130 and pBHR2 and their derivatives were likewise conjugated to *B. pseudomallei* by using *E. coli* S17-1 as the donor strain (Supplementary Table 1).

### Screening for *B. pseudomallei* Bp82 transposon mutants that do not produce a zone of inhibition on lawns of *Sphingobacterium* sp. ST4

*Sphingobacterium* sp. ST4 is naturally resistant to Km and this antibiotic was incorporated into the screening plates in order to ensure retention of Tn*Mod*-OKm’ in the Bp82 mutants. Individual Tn*Mod*-OKm’ mutants of Bp82 were picked from 150 x 15 mm polystyrene petri plates containing LB agar with adenine HCl (100 μg/ml), thiamine HCl (5 μg/ml), and Km (1,000 μg/ml) using sterile toothpicks. Prior to transfer, the agar medium was inoculated with *Sphingobacterium* sp. ST4 by submersing a sterile swab into a saturated LB broth culture and spreading it across the entire surface of the agar in back-and-forth motions. The agar plate was rotated 90° three times, and this process was repeated and the surface of the agar was allowed to dry in a class II microbiological safety cabinet prior to co-inoculation with *B. pseudomallei* Bp82 transposon mutants. The plates were incubated at 37°C for 1 to 2 days and screened for mutants that did not produce zones of inhibition (clearing) around the colonies. Potential Bp82 mutants were transferred to Ashdown Select Media agar plates (Ashdown, 1979) containing adenine HCl (100 μg/ml), thiamine HCl (5 μg/ml), carbenicillin (10 μg/ml) and polymyxin B (25 μg/ml) to select against *Sphingobacterium* sp. ST4 contamination. Approximately 6,000 transposon mutants were screened by this method.

### Construction of *B. pseudomallei* gene replacement mutants

Gene replacement experiments with *B. pseudomallei* were performed using the *sacB*-based vector pMo130 (Supplementary Table 1), as previously described (Burtnick et al., 2011; Hamad et al., 2009; Logue et al., 2009). Recombinant derivatives of pMo130 were electroporated into *E. coli* S17-1 (12.25 kV/cm) and conjugated with *B. pseudomallei* Bp82 or 1026b for 8 h. Pm was used to counterselect *E. coli* S17-1. The optimal conditions for the resolution of the *sacB* constructs were found to be LB agar lacking NaCl and containing 15% (wt/vol) sucrose with incubation at 37°C for 2 days. *B. pseudomallei* deletion mutants were identified by colony PCR using the primers flanking the deleted regions of the targeted genes. The mutants containing promoterless *lacZ* fusions to *hmqD* and *scmR* were identified by using a gene-specific primer and lacZ-dn2 (Supplementary Table 1).

### Bacterial competition assays

Thirty-nine unique environmental Gram-negative bacteria were initially assessed for their potential to form a zone of inhibition when grown as a bacterial lawn in the presence of Bp82. Briefly, each environmental bacterial isolate was grown in LB broth for 18 h, diluted 1:10 or 1:50 in PBS, and a sterile swab was used to spread the diluted culture across the entire surface of a LB agar in back-and-forth motions. The agar plate was co-inoculated with *B. pseudomallei* Bp82 in triplicate. The plates were incubated at 37°C for 1 to 2 days and screened for strains that produced zones of inhibition (clearing) around the Bp82 colonies and the inhibition zones were measured with a ruler. For those bacteria that formed zones of inhibition with Bp82, the same process was repeated with Bp82 *ΔhmqD* to identify environmental isolates that were inhibited in a 2-alkylquinolone-dependent manner. These bacteria were further assessed in a bacterial coculture competition assay.

A modified quantitative bacterial coculture competition assay (Hachani et al., 2013) was employed to assess the ability of *B. pseudomallei* to compete with the environmental Gram negative bacteria. Briefly, *B. pseudomallei* and environmental competitors were grown in LB broth at 37°C for 18 h, and three independent cultures of each strain were used for each competition assay performed. Two hundred microliters of each of the saturated cultures was pelleted by centrifugation, washed with sterile PBS, and diluted to ∼1 x 10^7^ CFU/ml, and 10 μl aliquots of each competitor were spotted onto the surface of LB agar or LB agar containing X-Gal. One hundred microliters of *B. pseudomallei* and of the environmental competitor were also combined and mixed, and a 20 μl aliquot of the 1:1 mixture was spotted onto the solid medium and incubated for 48 h at room temperature (RT). The remaining competition mixture was serially diluted in PBS, and 100–μl aliquots were spread onto LB agar or LB agar containing X-Gal to determine the input concentration of *B. pseudomallei* and environmental species present in the mixture. Following incubation, the bacteria present in each spot were resuspended in 1 ml of PBS using sterile swabs and serially diluted in PBS, and 100 μl aliquots were spread onto LB agar or LB agar containing X-Gal and incubated for 1 to 2 days at 37°C to determine the number of CFU present. The quantity of each competitor present in the competition mixture was assessed by enumerating the number of *B. pseudomallei* off-white colonies compared to the number of Gram-negative pigmented or blue colonies. The fold difference between the *B. pseudomallei*/environmental isolate ratio when the bacteria were grown alone relative to the *B. pseudomallei*/environmental isolate ratio when the bacteria were grown in mixed culture was used to establish the overall competitive index. For Gram-negative environmental strains that could not easily be differentiated from *B. pseudomallei* Bp82 by pigmentation or β-galactosidase production, selective agar medias were incorporated. R2A agar plates were utilized to select against Bp82 and LB agar plates with adenine HCl, thiamine HCl, Km, and Pm were used to select against Gram-negative competitors. Three independent pairs of cultures were performed for each *B. pseudomallei*-environmental isolate competition assay, and the results were recorded as the mean ± the standard deviation.

### β-galactosidase assays

*B. pseudomallei* strains harboring *hmqD*-*lacZ* and *scmR*-*lacZ* transcriptional fusions were inoculated into LB broth and grown overnight, diluted 1:100 in LB broth, and 1 ml aliquots were removed at 5 h and assayed for β-galactosidase activity as described previously (Miller, 1972). When indicated, synthetic *N*-octanoyl-L-homoserine lactone (C_8_-HSL; Sigma-Aldrich, St. Louis, MO), *N*-3-hydroxydecanoyl-L-homoserine lactone (3OHC_10_-HSL; Sigma-Aldrich, St. Louis, MO), and *N*-(3-hydroxy-octanoyl)-homoserine lactone (3OHC_8_-HSL; Cayman Chemical Company, Ann Arbor, MI)) were added to the culture medium of CM139 derivatives at a final concentration of 5 μM. AHLs were resuspended in acidified ethyl acetate at 10 mg/ml and stored at −20°C.

### Quantification of quinolones in bacterial cultures

Overnight cultures of Bp82, Bp82 *ΔhmqD*, Bp82 *ΔscmR*, and CM139 (150 μl) were inoculated into 10 ml of LB medium in sterile 50 ml polypropylene tubes with screw caps (VWR). The caps of the tubes were loosely opened by a 180-degree turn and fixed in this position with tape to ensure equal oxygen supply. The cultures were incubated at 37°C and 180 rpm. The samples (1 ml) were collected at 1.5, 3.0, 4.5, 6.0, 7.5, 9.0, and 24 h of incubation, centrifuged at 4500 rpm for 10 min, and supernatants were sterile filtered. Three hundred microliters of culture supernatant were added to 1.5 ml glass vials (LABSOLUTE, Art. Nr. 7612960) with caps containing a PTFE membrane (LABSOLUTE, Art. Nr. 7623097) and 300 μl of ethyl acetate (EtOAc) was added and vortexed for 5 s. After separation of organic and water phases, 100 μl of the EtOAc layer were transferred via pipetting into mass spectrometry vials containing a glass insert (MACHEREY-NAGEL, Art. Nr. 702007). The EtOAc was evaporated by a gentle stream of nitrogen. For liquid chromatography with tandem mass spectrometry (LC-MS/MS) analysis, 100 μl of sample solvent (MeOH/H_2_O 1:1) was added into glass inserts and the residue redissolved. The experiment was performed in biological triplicates.

### Bacterial survival and replication within the RAW264.7 murine macrophage-like cell line

RAW264.7 cells were infected at a MOI of 10:1 bacteria/cell. After a 2 h incubation at 37°C with 5% CO_2_, the cells were washed with PBS (Thermo Fisher Scientific) and fresh medium containing 1 mg/ml Km was added to kill the extracellular bacteria. The infected RAW 264.7 cells were incubated for an additional 10 h and were lysed with 0.1% SDS at designated time points and the number of intracellular bacteria were enumerated by serial dilution and plating on LB agar. The experiment was performed in biological triplicates and the results were recorded as the mean ± the standard deviation.

### Bacterial virulence in BALB/c mice

Groups of 6–8 week old BALB/c mice (n = 10) were infected intraperitoneally with increasing doses of 10^3^–10^6^ CFU of *B. pseudomallei* 1026b or *B. pseudomallei* 1026b *ΔhmqD*. Bacteria were grown overnight in a 37°C shaker at 200 rpm in LB broth and the inoculum counts were verified by serial dilution and plated on LB agar. Infected animals were monitored several times a day for 28 days to evaluate for morbidity or mortality. At the completion of the experiment, spleens from three randomly selected survivors per group were homogenized, diluted in PBS, and spread onto Sheep Blood Agar to assess bacterial colonization of the spleen.

### Inhibition of quinolone production in *B. pseudomallei* Bp82

An overnight culture (45 μl) of *B. pseudomallei* Bp82 was inoculated into 3 ml of LB broth in a sterile 15 ml polypropylene centrifuge tube with a screw cap (VWR). Compound 4 (3 μl) was added from a DMSO stock, to reach its final concentration of either 1 or 0.1 μM. DMSO (3 μl) was added as a control. The caps of the tubes were loosely opened by a 180-degree turn and fixed in this position with tape to ensure equal oxygen supply. The cultures were incubated at 37°C, 180 rpm, for 6.5 h and reached a 1.28 average OD_600_ value. After the incubation, the samples (3 ml) were centrifuged at 4500 rpm for 10 min, and supernatants were sterile filtered. Three hundred microliters of culture supernatant were added in 1.5 ml glass vials (LABSOLUTE, Art. Nr. 7612960) with caps containing a PTFE membrane (LABSOLUTE, Art. Nr. 7623097) and 300 μl of EtOAc were added and vortexed for 5 s. After separation of organic and water phases, 100 μl of the EtOAc layer were transferred via pipetting into mass spec vials containing a glass insert (MACHEREY-NAGEL, Art. Nr. 702007). The EtOAc was evaporated by a gentle stream of nitrogen. For LC-MS/MS analysis, 100 μl of sample solvent (MeOH/H_2_O 1:1) was added into glass inserts and the residue redissolved. The experiment was performed in biological triplicates.

### LC-MS/MS analysis

LC-MS/MS analysis was performed as described by Prothiwa et al. (Prothiwa et al., 2021) and quinolone standards were synthesized as described by Szamosvari et al. (Szamosvári et al., 2020). Ultra-high performance liquid chromatography was performed on a Vanquish™ UHPLC system (Thermo Fisher Scientific) using a Nucleodur C18 Gravity-SB 100 x 2 mm, 3 μm column (Macherey-Nagel). The flow rate was 0.5 ml min^–1^ and the column temperature was held at 40°C. The injection volume was 5 μl. Eluent A was 0.1% formic acid in water and eluent B was 0.1% formic acid in acetonitrile. The gradient was 20–100 % B in 10 min, 100 % B for 2 min, 100–20 % B in 1 min, and 20 % B for 2 min. MS/MS analysis was performed by TSQ^®^ Series II Quantis (Thermo Fisher Scientific) mass spectrometer. A heated electrospray ionization (HESI-II probe, Thermo Scientific) was used as an ion source. In the optimized conditions the ion spray voltage was 3500 V, vaporizer temperature 300°C, ion transfer tube temperature 380°C, sheath gas pressure 60 psi, ion sweep gas pressure 2 psi, and aux gas 10 psi. The fragmentation pattern of quinolone standards was acquired in a Product Ion Scan mode using a fixed collision energy of 30 V to fragment the corresponding precursor ion before recording the fragments in a mass range of m/z 130–350. Quinolones we quantified in Selected Reaction Monitoring scan mode. MS/MS spectra were acquired in a positive mode. The software Quan Browser Thermo Xcalibur was used for quantitative analysis.

### Statistical analyses

Three independent pairs of cultures were performed for each coculture competition assay and the results were recorded as the mean ± the standard deviation. A statistical analysis of the results involved ANOVA and pairwise group comparisons. The β-galactosidase assays, the production of Δ^2^-MNQ, MNQ, and Δ^2^-MNQNO, and the RAW 264.7 intracellular assays were conducted in triplicate and were recorded as the mean ± the standard deviation. The 1026b LD_50_ in mice was estimated under a Probit Model with Log transformation of the dose variable. The survival rates at selected time points were compared by Fisher exact test and the times to death (TTD) were analyzed by Log-rank test for the pairwise comparison between the challenged groups. Analysis was implemented using SAS version 9.4.

## Results

### Isolation and characterization of Gram-negative environmental bacteria for competition studies

**FIGURE 1:**
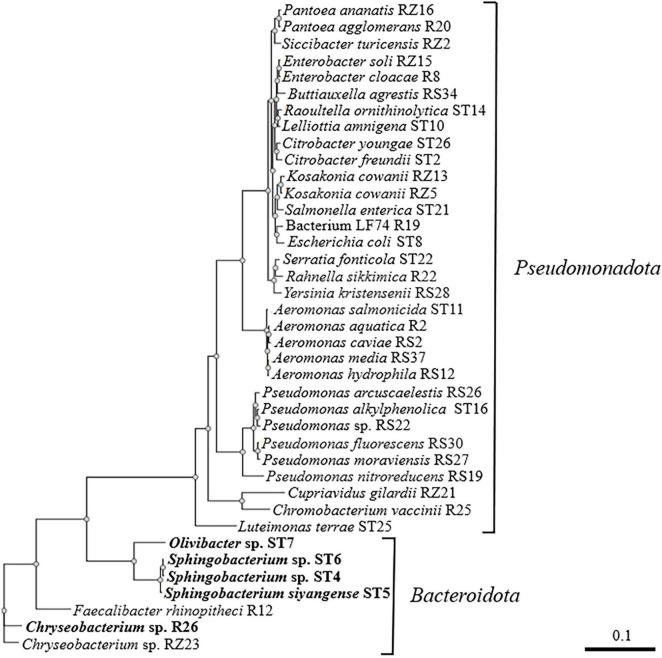
Phylogenetic tree based on partial 16S rRNA sequences of Gram-negative bacterial isolates from river water (R), river sediment (RS), stream water (ST), and rhizosphere (RZ) samples. NGPhylogeny.fr was used to build the tree in “FastME/OneClick” mode and it utilized MAFFT for multiple alignment, BMGE for automatic alignment curation, FastME for tree inference, and Newick Display for tree rendering (Lemoine et al., 2019). The bacterial strains that are inhibited by *B. pseudomallei* Bp82 in both zone of inhibition and coculture competition assays are shown in bold font. The scale bar indicates the number of substitutions per site.

*B. pseudomallei* is a saprophytic organism that encounters microbial competitors in the soil, water, and rhizosphere in many regions around the world (Inglis and Mayo, 2012; Kaestli et al., 2012; Limmathurotsakul et al., 2012; Petras et al., 2023). In a previous study, environmental bacteria were isolated from diverse sources, including soil (S), river water (R), river sediment (RS), stream water (ST), and the rhizosphere (RZ), and Gram-positive bacteria were evaluated for their ability to compete with *B. pseudomallei* (Mou et al., 2021). A similar strategy was employed here to isolate additional environmental bacteria and examine the competitive interactions between Gram-negative isolates and *B. pseudomallei*. Such experiments cannot replicate the natural environment of the competitors but can provide information about the bacterial factors that facilitate competition under specific laboratory conditions. Bacteria that could be easily distinguished from *B. pseudomallei* colonies on solid medium due to colony morphology, pigmentation, and/or β-galactosidase production were isolated from all environmental sources except soil. In addition, multiple bacterial colonies that could not be easily differentiated from *B. pseudomallei* were also retained and further characterized. When combined with the environmental bacteria isolated previously (Mou et al., 2021), a total of 39 distinct Gram-negative bacterial species were identified. Figure 1 shows the evolutionary relationships of these bacteria and demonstrates that there were 32 isolates in the phylum *Pseudomonadota* and 7 isolates in the phylum *Bacteroidota* (Oren and Garrity, 2021).

An initial antimicrobial activity assay was utilized to identify environmental bacteria that produced a zone of inhibition (clearing) when grown as an agar lawn in the presence of *B. pseudomallei*. Strain Bp82 formed a zone of clearing on agar lawns of 10 environmental Gram-negative bacteria, including *Aeromonas hydrophila* RS12, *Pseudomonas arcuscaelestis* RS26, *Pseudomonas moraviensis* RS27, *Faecalibacter rhinopitheci* R12, *Chryseobacterium* sp. R26, *Sphingobacterium* sp. ST4, *Sphingobacterium siyangense* ST5, *Sphingobacterium* sp. ST6, *Olivibacter* sp. ST7, and *Chryseobacterium* sp. RZ23 (Table 1). By comparison, 29/39 (∼74%) of the Gram-negative bacteria did not produce a zone of inhibition when grown in the presence of Bp82 (data not shown).

**TABLE 1:** In vitro competition assays used to identify environmental bacteria inhibited by *B. pseudomallei*.

	Formed a zone of growth inhibition on an agar lawn^a^		Inhibited in a coculture competition assay^b^	
**Strain (phylum)**	**Bp82**	**Bp82** ***ΔhmqD***	**Bp82**	**Bp82 *ΔhmqD***
*Aeromonas hydrophila* RS12 (*Pseudomonadota*)	Yes	No	Yes	Yes
*Pseudomonas arcuscaelestis* RS26 (*Pseudomonadota*)	Yes	No	No	No
*Pseudomonas moraviensis* RS27 (*Pseudomonadota*)	Yes	No	No	ND^c^
*Faecalibacter rhinopitheci* R12 (*Bacteroidota*)	Yes	No	No	ND
*Chryseobacterium* sp. R26 (*Bacteroidota*)	Yes	No	Yes	No
*Sphingobacterium* sp. ST4 (*Bacteroidota*)	Yes	No	Yes	No
*Sphingobacterium siyangense* ST5 (*Bacteroidota*)	Yes	No	Yes	No
*Sphingobacterium* sp. ST6 (*Bacteroidota*)	Yes	No	Yes	No
*Olivibacter* sp. ST7 (*Bacteroidota*)	Yes	No	Yes	No
*Chryseobacterium* sp. RZ23 (*Bacteroidota*)	Yes	No	No	ND

^a^Environmental bacteria were spread onto the surface of agar plates and Bp82 or Bp82 *ΔhmqD* were inoculated at a single point with a sterile toothpick. Plates were incubated at 37°C for 1 to 2 days and screened for strains that produced zones of inhibition (clearing) around the Bp82 or Bp82 *ΔhmqD* colonies.

^b^A modified quantitative bacterial coculture competition assay (Hachani et al., 2013) was employed to assess the ability of *B. pseudomallei* to compete with the environmental Gram negative bacteria (see Materials and Methods for details).

^c^Not determined.

### Tn*Mod*-OKm’ mutagenesis of *B. pseudomallei* Bp82 identifies two distinct loci required for inhibiting the growth of *Sphingobacterium* sp. ST4

**FIGURE 2:**
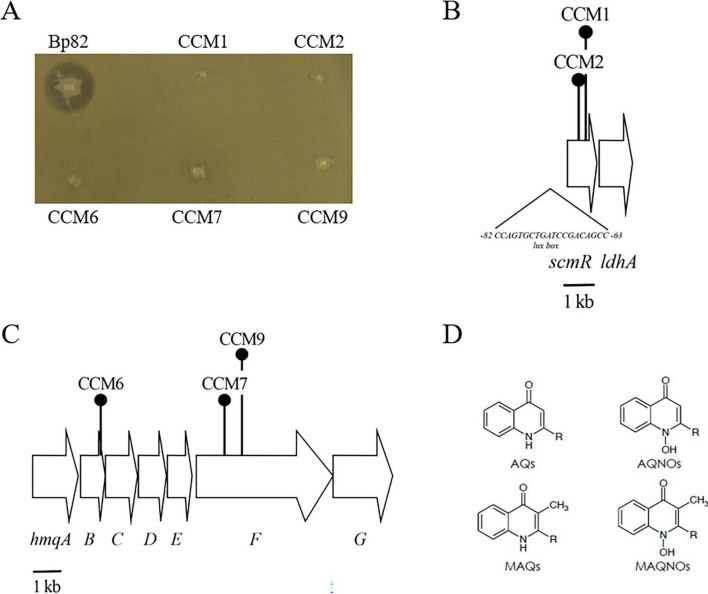
The *B. pseudomallei hmqA-G* locus and the *scmR* gene are required for antimicrobial activity against *Sphingobacterium* sp. ST4. **(A)** Bp82 produces a zone of inhibition on a lawn of ST4, but CCM1, CCM2, CCM6, CCM7, and CCM9 are unable to produce such zones. The inoculated LB agar plate was incubated for 24 h at 37°C. **(B)** Genetic map of the *scmR* and *ldhA* genes, locus tags BP1026B_I0582 - BP1026B_I0581, in *B. pseudomallei* 1026b. The location of a putative *lux* box at −82 to −63 nucleotides upstream of the *scmR* start codon is also shown (Le Guillouzer et al., 2020). The *scmR* gene encodes a LTTR and *ldhA* encodes a putative lactate dehydrogenase. A 1 kilobase (kb) scale is shown at the bottom. **(C)** Genetic map of the *hmqA-G* gene cluster, locus tags BP1026B_II0535 - BP1026B_II0541, in *B. pseudomallei* 1026b. The location and direction of transcription of genes are represented by arrows. The gene products of this locus are required for the biosynthesis of AQs and MAQs. The locations of Tn*Mod*-OKm’ insertions in CCM6, CCM7, and CCM9 are shown schematically by round-top push pins. The unlinked *hmqL* gene required for generating AQNOs and MAQNOs (Klaus et al., 2020b), locus tag BP1026B_II2272 in 1026b, is not shown. **(D)** Chemical structure of AQs, AQNOs, MAQs, and MAQNOs encoded by the *B. pseudomallei hmqA-G* locus. The R represents the presence of unsaturated or saturated alkyl groups of seven to eleven carbons at the 2-position.

Mou et al. developed a Tn*Mod*-OKm’ screen to identify *B. pseudomallei* mutants that did not inhibit the growth of the Gram-positive bacterium *Neobacillus bataviensis* S4 (Dennis and Zylstra, 1998; Mou et al., 2021). A similar strategy was used in this study to identify Bp82 mutants that could not form a zone of clearing on the Gram-negative bacterium *Sphingobacterium* sp. ST4. Approximately 6,000 Bp82 transposon mutants were screened, and five, termed CCM1, CCM2, CCM6, CCM7, and CCM9, did not exhibit antimicrobial activity against ST4 (Figure 2A). CCM1 and CCM2 contained transposon insertions in *scmR* (Figure 2B), a LTTR that serves as a global regulator of BGCs in *B. pseudomallei* and *B. thailandensis* (Klaus et al., 2018; Mao et al., 2017). The sites of Tn*Mod*-OKm’ insertions in CCM6, CCM7, and CCM9 were mapped to the *hmqA-G* locus (Figure 2C), which encodes the biosynthetic enzymes involved in the production of AQs, AQNOs, MAQs and MAQNOs in *B. pseudomallei*, *B. thailandensis*, and some members of the *Burkholderia cepacia* complex (Diggle et al., 2006; Klaus et al., 2020a; Klaus et al., 2020b; Mou et al., 2021; Piochon et al., 2020; Szamosvári et al., 2020; Vial et al., 2008; Figure 2D). Bp82 strains with defined deletion mutations in *hmqD* and *scmR*, Bp82 *ΔhmqD* and Bp82 *ΔscmR* (Supplementary Table 1), also did not produce zones of inhibition on a ST4 lawn (Supplementary Figure 1). The full-length *scmR* gene was cloned into the broad-host-range vector pBHR2 and conjugated to Bp82 *ΔscmR* in an attempt to complement the *ΔscmR* mutation. The result demonstrated that Bp82 *ΔscmR* (pBHR2-*scmR*) formed a zone of clearing on a ST4 lawn, but Bp82 *ΔscmR* (pBHR2) did not (Supplementary Figure 2). The fact that Bp82 *ΔscmR* could be complemented by providing *scmR in trans* suggests that the *ΔscmR* mutation does not have a polar effect on downstream genes, including *ldhA* (Figure 2B). Importantly Bp82 *ΔhmqD* could also be complemented by providing full length *hmqD* on pBHR2 (Mou et al., 2021). Further experiments were conducted with the other Gram-negative bacteria that produced zones of inhibition on agar lawns with Bp82 and the results indicated that formation of inhibition zones were strictly dependent on a functioning copy of *hmqD* (Table 1). Taken together, the results show the *hmqA-G* operon and the regulatory gene *scmR* are involved in the ability of Bp82 to form zones of clearing on agar lawns of ten distinct environmental Gram-negative species.

### *B. pseudomallei* outcompetes bacteria in the phylum *Bacteroidota* in zone of inhibition and coculture competition assays

**FIGURE 3:**
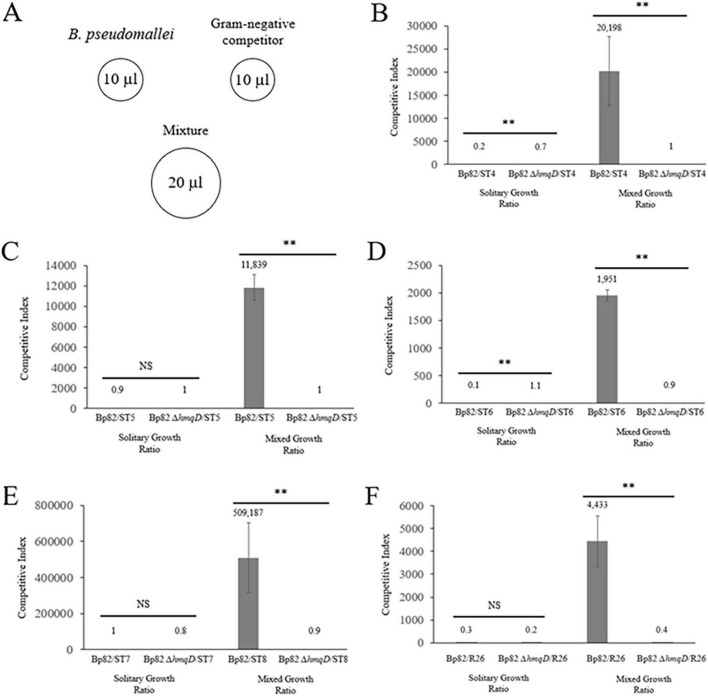
*B. pseudomallei* outcompetes environmental Gram-negative bacteria in the phylum *Bacteroidota* in an *hmqD*-dependent manner. **(A)** Schematic illustration of coculture competition assays performed with *B. pseudomallei* Bp82 and **(B)** *Sphingobacterium* sp. ST4, **(C)** *Sphingobacterium* sp. ST5, **(D)** *Sphingobacterium* sp. ST6, **(E)** *Olivibacter* sp. ST7, and **(F)** *Chryseobacterium* sp. R26. The fold difference between the *B. pseudomallei*/environmental isolate ratio when the bacteria were grown alone (solitary growth ratio) or in mixed culture (mixed culture ratio) was used to establish the competitive indexes. The bacteria were incubated at RT for 48 h on solid medium and the surviving competitors were quantitated. Three independent pairs of cultures were performed for each competition assay and the results were recorded as the mean ± the standard deviation. A statistical analysis of the results involved ANOVA and pairwise group comparisons. NS, not significant; **, *P* < 0.0001.

As demonstrated above, Bp82 was able to form a zone of clearing on 100% of the bacteria in the *Bacteroidota* phylum and 9% of the bacteria in the *Pseudomonadota* phylum (Figure 1 and Table 1). To further assess the interaction of *B. pseudomallei* with the 10 environmental Gram-negative bacteria that were inhibited in a *hmqD*-dependent fashion in coculture competition assays, a quantitative bacterial competition assay was employed (Hachani et al., 2013). Briefly, *B. pseudomallei* and environmental competitors were grown in liquid broth and 10 μl aliquots of each were spotted onto the surface of agar media (Figure 3A). Twenty-microliter aliquots of 1:1 mixtures of *B. pseudomallei* and environmental competitors were also spotted onto the solid medium and incubated for 48 h at RT. The competition results were quantitated by resuspending the bacterial spots in PBS, performing serial dilutions, and spreading aliquots onto agar plates. The quantity of bacterial competitors present in the competition mixtures was assessed by enumerating the number of *B. pseudomallei* off-white colonies and comparing it to the number of Gram-negative pigmented or blue colonies. For those environmental bacteria with colony morphotypes that could not be readily differentiated from *B. pseudomallei*, selective agar media was used to quantitate the competitors (see Materials and Methods). The Bp82/environmental strain solitary growth ratio was compared to the Bp82/environmental strain mixed growth ratio and used to assess the fold difference between the two growth indices. The results of quantitative coculture competition assays between *B. pseudomallei* Bp82 and the environmental strains are shown in Table 1. *B. pseudomallei* Bp82 outcompeted *Sphingobacterium* sp. ST4, *S. siyangense* ST5, *Sphingobacterium* sp. ST6, *Olivibacter* sp. ST7, and *Chryseobacterium* sp. R26 and the results were dependent on an intact copy of the *hmqD* gene as the Bp82 *ΔhmqD* mutant was unable to compete with these Gram-negative species (Figures 3B–F). Interestingly, *F. rhinopitheci* R12, *Chryseobacterium* sp. RZ23, *P. arcuscaelestis* RS26, and *P. moraviensis* RS27 were inhibited by Bp82 when grown on agar lawns, but were not inhibited by Bp82 in coculture competition assays (Table 1 and Supplementary Figure 3). These species may possess factors that allow them to effectively compete with *B. pseudomallei* when grown in coculture. Both Bp82 and Bp82 *ΔhmqD* outcompeted *A. hydrophila* RS12, indicating that the inhibition of this bacterium requires other factors than the natural quinolones produced by the *B. pseudomallei hmqA-G* locus. The results demonstrate that only Gram-negative bacteria in the phylum *Bacteroidota* were inhibited in a *hmqD*-dependent fashion in both zone of inhibition and coculture competition assays (Figure 1).

### Expression of *hmqD* in *B. pseudomallei* is dependent on ScmR, a LTTR that is positively regulated by quorum sensing

**FIGURE 4:**
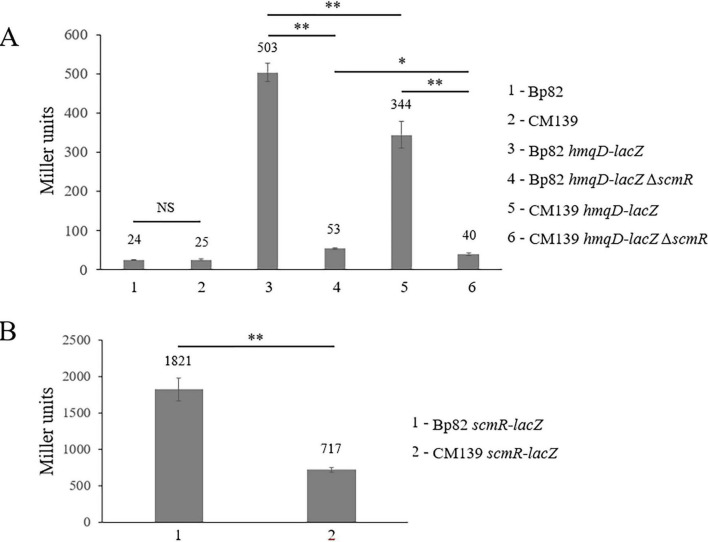
Expression of the *B. pseudomallei hmqD* gene is positively regulated by ScmR which is activated by quorum sensing. β-galactosidase production by *B. pseudomallei* strains containing chromosomal promoterless *lacZ* reporter fusions to assess *hmqD* **(A)** and *scmR* **(B)** expression during mid-logarithmic phase growth in LB broth. β-galactosidase was measured spectrophotometrically and the Miller units were assessed as described (Miller, 1972). Each numerical value for β-galactosidase production is the mean of experiments performed on at least three separate occasions ± standard deviation (bars). Pairwise treatment groups were compared by negative binomial generalized linear model and no multiplicity adjustment was applied. NS, not significant; *, *P* < 0.05; **, *P* < 0.0001.

Previous studies have examined the expression of *hmqA-G* and *scmR* in *Burkholderia*, with most focusing exclusively on *B. thailandensis* (Coulon et al., 2019; Le Guillouzer et al., 2020; Klaus et al., 2018; Majerczyk C. et al., 2014; Majerczyk C. D. et al., 2014; Mao et al., 2017; Martinez et al., 2020). Given the importance of these two loci for the competitive interaction of *B. pseudomallei* with environmental bacteria, the expression of these genes was further evaluated here. The promoterless *lacZ* gene was fused to *hmqD* and *scmR* in *B. pseudomallei* Bp82 and CM139, a quorum sensing (QS) deficient mutant (Supplementary Table 1). CM139 is a Bp82 derivative with deletions in all three of the AHL synthase genes (*bpsI1* to *–3*). *B. pseudomallei* produces three *N*-acyl homoserine lactones (AHLs), C_8_-HSL, 3OH-C_10_-HSL, and 3OH-C_8_-HSL, that are involved in population density-dependent gene expression (Fuqua et al., 2001; Majerczyk C. D. et al., 2014). Figure 4A shows that Bp82 and CM139 only produce background levels of β-galactosidase activity, 24 and 25 Miller units, respectively, because *B. pseudomallei* strains do not naturally possess this enzyme. Beta-galactosidase assays were conducted with Bp82 *hmqD*-*lacZ* and CM139 *hmqD*-*lacZ* and the results showed that the transcription of *hmqD* was decreased by approximately one third in the absence of AHLs (Figure 4A). By comparison, the expression of *hmqD* in Bp82 *hmqD*-*lacZ* and CM139 *hmqD*-*lacZ* was decreased by nine- to ten-fold in the absence of *scmR* (Figure 4A). The results show that QS and ScmR are both important for maximal transcription of the *hmqA-G* operon, but demonstrate that ScmR plays a more important role in the positive regulation of this locus than QS. When Bp82 *scmR*-*lacZ* and CM139 *scmR*-*lacZ* were subjected to β-galactosidase assays, the transcription of *scmR* was decreased by 60% in the absence of AHLs (Figure 4B). Similarly, Klaus et al. found that *scmR* expression in CM139 was three-fold lower than in Bp82 (Klaus et al., 2018). Taken together, the results indicate that QS positively regulates *scmR* transcription and ScmR subsequently activates transcription of the *hmqA-G* locus (Figure 4).

**FIGURE 5:**
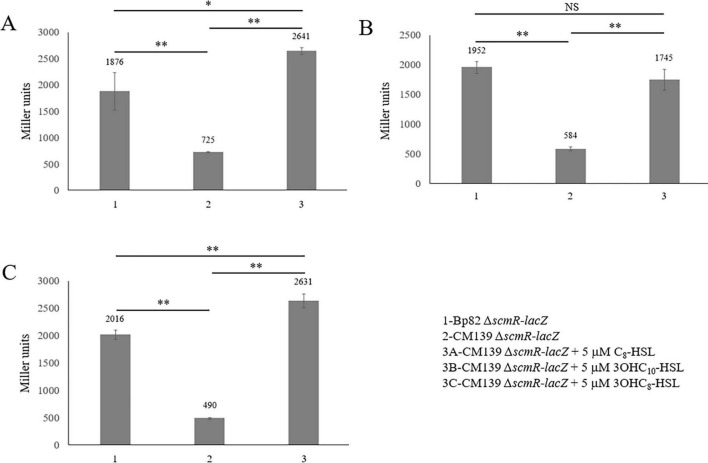
Addition of *B. pseudomallei* homoserine lactones to growth media increased the expression of *scmR-lacZ* in the quorum sensing-deficient strain CM139. β-galactosidase production by *B. pseudomallei* CM139 *scmR-lacZ* was assessed during mid-logarithmic phase growth in LB broth with HSLs exogenously added to a final concentration of 5 μM. C_8_-HSL **(A)**, 3OHC_10_-HSL **(B)**, and 3OHC_8_-HSL **(C)**. Each numerical value for β-galactosidase production is the mean of experiments performed on at least three separate occasions ± standard deviation (bars). Pairwise treatment groups were compared by negative binomial generalized linear model and no multiplicity adjustment was applied. *, *P* < 0.005; **, *P* < 0.0001.

The *B. pseudomallei scmR* gene contains a *lux* box 63–82 bp upstream of the ATG start codon (Figure 2B) which supports the fact that this gene is regulated by QS (Klaus et al., 2018; Le Guillouzer et al., 2020). Each of the three *B. pseudomallei* AHLs were added to CM139 *scmR*-*lacZ* to determine if commercially acquired C_8_-HSL, 3OH-C_10_-HSL, or 3OH-C_8_-HSL could activate the transcription of *scmR*. The AHLs were individually added to CM139 *scmR*-*lacZ* cultures at a final concentration of 5 μM and β-galactosidase assay results were compared to Bp82 *scmR*-*lacZ* and CM139 *scmR*-*lacZ* cultures grown without AHLs (Figure 5). The AHL C_8_-HSL, produced by BpsI1 AHL synthase, completely restored *scmR* transcription in CM139 *scmR*-*lacZ* to the expression levels of Bp82 *scmR*-*lacZ* (Figure 5A). In fact, CM139 *scmR*-*lacZ* with added C_8_-HSL resulted in approximately 30% higher *scmR* transcription than in Bp82 *scmR*-*lacZ*. The AHL produced by BpsI2 AHL synthase, 3OH-C_10_-HSL, also restored *scmR* expression levels in CM139 *scmR*-*lacZ* to those present in Bp82 *scmR*-*lacZ* (Figure 5B). The BpsI3 AHL synthase product, 3OH-C_8_-HSL, also increased *scmR* transcription in CM139 *scmR*-*lacZ* to levels that were approximately 23% higher than in Bp82 *scmR*-*lacZ* (Figure 5C). The concentration of AHLs added to the CM139 *scmR*-*lacZ* growth medium was above physiological concentrations, but allowed for saturation of the three *B. pseudomallei* QS systems (Majerczyk C. D. et al., 2014). The elevated levels of C_8_-HSL and 3OH-C_8_-HSL in the culture medium may be responsible for the increased transcription of *scmR* in CM139 *scmR*-*lacZ* relative to Bp82 *scmR*-*lacZ*. The results show that all three AHLs stimulate *scmR* expression in *B. pseudomallei* (Figure 5) which is consistent with experiments conducted in *B. thailandensis* (Majerczyk C. et al., 2014).

### Production of 2-alkylquinolone derivatives by Bp82 is modulated by QS and is dependent on *hmqA-G* and *scmR*

**FIGURE 6:**
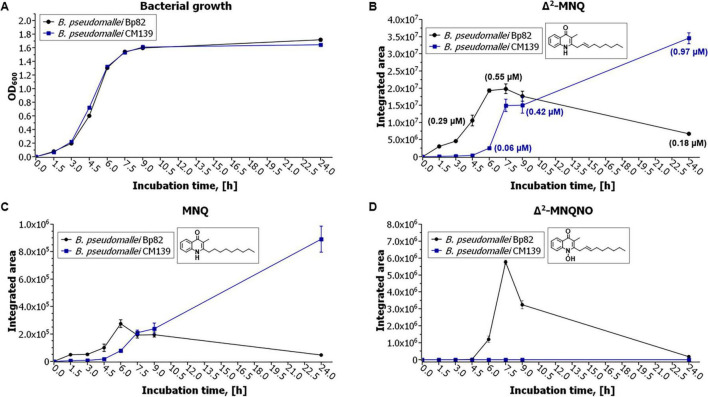
Growth and production of Δ^2^-MNQ, MNQ, and Δ^2^-MNQNO by *B. pseudomallei* Bp82 and CM139. **(A)** Bacterial growth curves of Bp82 (black) and CM139 (blue). The production levels of Δ^2^-MNQ **(B)**, MNQ **(C)**, and Δ^2^-MNQNO **(D)** by Bp82 (black) and CM139 (blue) during growth at 37°C for 24 h. Mean values are given for biological triplicates with the error bars representing the population standard deviation. The only time point that not statistically significant (*p* ≤ 0.05) for **(B)** was 9 h and for **(C)** was 7.5 h and 9 h.

LC-MS/MS analysis was performed on culture supernatants of Bp82, Bp82 *ΔhmqD*, Bp82 *ΔscmR*, and CM139 to assess the production of AQ, AQNO, MAQ, and MAQNO using the corresponding synthetic standards. Table 2 shows that Bp82 produced a variety of AQ and MAQ derivatives and the results were consistent with LC-MS/MS experiments previously described for *B. pseudomallei* (Diggle et al., 2006; Mou et al., 2021; Vial et al., 2008). It is important to emphasize that the 2-alkylquinolone molecules that possessed unsaturated alkyl chains contained a double bond at position 2′ rather than position 1′, as previously described (Mou et al., 2021). Bp82 *ΔhmqD* and Bp82 *ΔscmR* did not produce any AQ derivatives (Table 2) which confirms the importance of these compounds for *B. pseudomallei* competition with environmental Gram-negative bacterial species in the phylum *Bacteroidota* (Figures 2, 3, and Supplementary Figure 1). *B. pseudomallei* CM139 produced AQs and MAQs; however, compounds with *N*-oxide groups were not detected (Table 2). A further analysis demonstrated that the growth rates of Bp82 and CM139 in LB broth were nearly identical (Figure 6A) and the production levels of Δ^2^-MNQ, MNQ, and Δ^2^-MNQNO were assessed by LC-MS/MS over a 24 h period of growth (Figures 6B–D). All three metabolites were maximally produced by Bp82 during the late logarithmic to early stationary phase of growth followed by a steady decrease in production throughout the stationary phase (Figures 6B–D). The production of Δ^2^-MNQ and MNQ followed a similar trend in CM139 until the late logarithmic stage of growth when the production of these compounds continued to rise, rather than decrease, throughout the stationary phase (Figures 6B–C). As mentioned above, no Δ^2^-MNQNO was produced by CM139 during any stage of growth. The LC-MS/MS experiments support the bacterial competition and gene expression results and further demonstrate that the production of AQs, AQNOs, MAQs, and MAQNOs are dependent on QS, ScmR, and the products of the *hmqA-G* locus. The data also suggest that the unlinked gene *hmqL*, encoding a FAD-dependent monooxygenase required for the synthesis of AQNOs and MAQNOs, may also be regulated in a QS-dependent fashion (see below also).

**TABLE 2:** Production of AQ and MAQ derivatives following growth in LB broth at 37°C for 7 h.

Strain	AQs^a^	MAQs^b^
Bp82	HHQ, HQNO, NQ, NQNO, Δ^2^-NQ	MNQ, MNQNO, MHQ, Δ^2^-MNQ, Δ^2^-MNQNO
Bp82 *ΔhmqD*	ND^c^	ND
Bp82 *ΔscmR*	ND	ND
CM139	HHQ, NQ	MNQ, Δ^2^-MNQ

^a^HHQ, 2-heptyl-2-alkylquinolone; HQNO, 2-heptyl-4-hydroxyquinoline-*N*-oxide; NQ, 2-nonyl-4(1*H*)-quinolone; NQNO, 2-nonyl-4(1*H*)-quinolone *N*-oxide; Δ^2^-NQ, unsaturated *trans*-Δ^2^-2-nonyl-4(1*H*)-quinolone.

^b^MNQ, 3-methyl-2-nonyl-4(1*H*)-quinolone; MNQNO, MNQ *N*-oxide; MHQ, 3-methyl-2-heptyl-4-hydroxyquinoline; Δ^2^-MNQ, unsaturated *trans*-Δ^2^-3-methyl-2-nonyl-4(1*H*)-quinolone; Δ^2^-MNQNO, Δ^2^-MNQ *N*-oxide.

^c^ND, not detected.

### *B. pseudomallei* 1026b *ΔhmqD* exhibits reduced intracellular replication in RAW264.7 cells and diminished virulence in the BALB/c animal model of melioidosis

**FIGURE 7:**
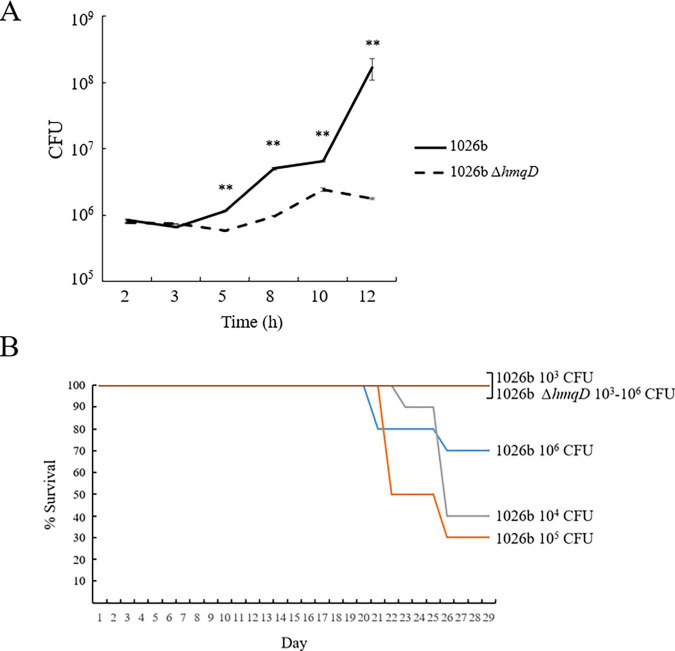
*B. pseudomallei* 1026b *ΔhmqD* exhibits reduced intracellular replication in RAW264.7 cells and reduced virulence in the BALB/c animal model of melioidosis. **(A)** RAW264.7 cells were infected with 1026b and 1026b *ΔhmqD* at a MOI of 10 and intracellular bacteria were enumerated for 12 h post infection. Each numerical value for CFU is the mean of experiments performed on at least three separate occasions ± standard deviation (bars). Pairwise treatment groups were compared by negative binomial generalized linear model. No multiplicity adjustment was applied. **, *P* < 0.0001. **(B)** Ten BALB/b mice were infected intraperitoneally with 10^3^–10^6^ CFU of 1026b and 1026b *ΔhmqD* and evaluated for morbidity and mortality for 28 days. The survival rates of mice that received 1026b *ΔhmqD* were greater than those that received 1026b, with *p* = 0.0108 at 10^4^ CFU and *p* = 0.0031 at 10^5^ CFU, respectively. The log-rank test *p*-value of Time to Death (TTD) for 1026b versus 1026b *ΔhmqD* was significant at 10^4^ CFU (*p* = 0.0046) and 10^5^ CFU (*p* = 0.0012), but not significant at 10^3^ CFU and 10^6^ CFU (*p* = 0.0669).

Aiosa et al. recently demonstrated that *B. thailandensis* produced AQ derivatives when grown inside RAW264.7 murine macrophage cells, but the importance of these compounds for replication and/or survival in this niche was not assessed (Aiosa et al., 2022). The *ΔhmqD* mutation was introduced into *B. pseudomallei* 1026b (Supplementary Table 1), the virulent parental strain of Bp82, and used to infect RAW264.7 cells. Figure 7A shows that survival and replication of 1026b and 1026b *ΔhmqD* were not significantly different during the first 3 h of infection, but by 12 h post infection there was a 94-fold reduction in the number of intracellular 1026b *ΔhmqD* bacteria relative to 1026b. This result indicates that AQ derivatives are required for optimal replication inside RAW264.7 cells, but the mechanism by which this occurs is currently unknown.

*B. pseudomallei* 1026b and 1026b *ΔhmqD* were also examined for their relative virulence in the BALB/c mouse model of infection (Figure 7B). Groups of 10 mice were infected via the intraperitoneal route with 10^3^, 10^4^, 10^5^, and 10^6^ CFU of 1026b and 1026b *ΔhmqD* and monitored for 28 days. All mice that received 1026b *ΔhmqD* survived during the period of observation, but morbidity and mortality was pronounced in animals that received 10^4^, 10^5^, and 10^6^ CFU of 1026b (Figure 7B). These mice exhibited noticeable reduced grooming and rough fir at two to three weeks postinfection, but all animals infected with 1026b *ΔhmqD* exhibited a normal appearance. The spleens from animals surviving challenge with 1026b contained > 10^5^ CFU/g at the end of the experiment. By comparison, the spleens from mice challenged with 1026b *ΔhmqD* harbored no detectable CFU, indicating complete immunological clearance of the mutant strain by day 28. The LD_50_ of 1026b and 1026b *ΔhmqD* was determined to be 3 x 10^5^ CFU and > 1 x 10^6^ CFU, respectively. This represents the first time that a mammalian model of infection has been utilized to demonstrate that the *hmqA-G* locus is important for *B. pseudomallei* virulence. Further studies will be required to reveal if there is a direct link between the 1026b *ΔhmqD* macrophage replication defect and the decreased virulence *in vivo* or if there is no connection between these biological phenomena.

### A synthetic chemical inhibitor of the central quinolone biosynthesis enzyme HmqD blocks MAQ and MAQNO production in *B. pseudomallei* Bp82

**FIGURE 8:**
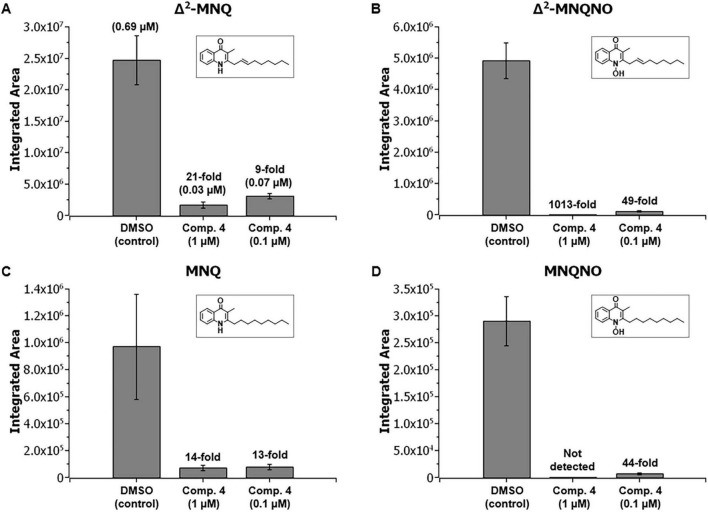
*B. pseudomallei* Bp82 production of MAQs and MAQNOs was inhibited by a compound that specifically targets the quinolone biosynthesis enzyme HmqD. Δ^2^-MNQ **(A)**, Δ^2^-MNQNO **(B)**, MNQ **(C)**, and MNQNO **(D)** production inhibition in Bp82 after treatment with 1-(2-amino-5-iodophenyl)-2-chloroethan-1-one, compound 4 (Prothiwa et al., 2021) at 1 and 0.1 μM final concentration. Fold change is calculated with respect to DMSO control. Mean values are given for biological triplicates with the error bars representing the population standard deviation.

Prothiwa et al. previously developed chemical inhibitors of the *B. ambifaria* HmqD enzyme binding covalent into the active site that were able to prevent quinolone production in this species at micromolar concentrations (Prothiwa et al., 2021). One of these synthetic inhibitors, 1-(2-amino-5-iodophenyl)-2-chloroethan-1-one (compound 4), was further examined here to see if it could also prevent quinolone production in *B. pseudomallei* Bp82. Bacterial broth cultures were grown aerobically with compound 4 at final concentrations of 1 and 0.1 μM and the filtered supernatants were assessed for quinolone production via LC-MS/MS analysis (Figure 8). The fold change of quinolone production was calculated with respect to a DMSO control. Compound 4 significantly inhibited the production of Δ^2^-MNQ (Figure 8A), Δ^2^-MNQNO (Figure 8B), MNQ (Figure 8C), and MNQNO (Figure 8D) in *B. pseudomallei* at both concentrations; however, all quinolones were inhibited more efficiently by the higher concentration except MNQ. Given the fact that the *hmqA-G* locus is important for virulence in the host (Figure 7B), the inhibition of quinolone production by chemicals like compound 4 could represent a novel therapeutic strategy against *B. pseudomallei*. While a preliminary study with compound 4 indicated that it is cytotoxic for HepG2 cells (data not shown), the future development of nontoxic HmqD inhibitors should allow their evaluation as anti-infectives in the murine model of melioidosis.

## Discussion

*B. pseudomallei* is an opportunistic pathogen that resides in soil and water in tropical and subtropical regions and humans are infected by inhalation or contact with contaminated environmental sources (Inglis and Mayo, 2012; Limmathurotsakul et al., 2016; Limmathurotsakul et al., 2012; Meumann et al., 2024). The inherent ability of *B. pseudomallei* to persist in the environment is a key reason why melioidosis continues to be an important infectious disease around the world. Mou et al. demonstrated that *B. pseudomallei* AQ derivatives are required for optimal competitive fitness with environmental Gram-positive bacteria and the results presented here show that these metabolites are also important for competition with Gram-negative bacteria in the *Bacteroidota* phylum (Mou et al., 2021). Bacteria in the *Pseudomonadota* phylum employ UQ for aerobic respiration, but Gram-positive bacteria and members of the *Bacteroidota* phylum only utilize DMK and/or MK for aerobic respiration (Marreiros et al., 2016; Schoepp-Cothenet et al., 2013; Zhi et al., 2014). Isoprenoid quinones such as UQ, DMK, and MK are lipid-soluble electron carriers in the cytoplasmic membrane and function as mobile redox carriers in the respiratory chain. The two major types of isoprenoid quinones are benzoquinones (UQ, plastoquinone, and rhodoquinone) and naphthoquinones (MK and DMK) (Abby et al., 2020; Nowicka and Kruk, 2010). They are composed of a hydrophilic head group and an apolar isoprenoid side chain that can vary in length and saturation. Naphthoquinones are the oldest quinones that first appeared when the earth contained a more reducing atmosphere. Benzoquinones, on the other hand, arose after the appearance of photosynthetic organisms and the corresponding increase in oxygen levels. They have a higher midpoint redox potential than naphthoquinones and are ideal for aerobic metabolism. Many members of the *Pseudomonadota* phylum, like *E. coli*, contain UQ, DMK, and MK, but use UQ for aerobic respiration and DMK and MK for anaerobic respiration. Gram-positive bacteria and Gram-negative bacteria in the *Bacteroidota* phylum do not synthesize UQ and must use DMK and MK for aerobic respiration. It has been proposed that *Burkholderia* MAQNOs structurally resemble the MK/MKH_2_ redox couple that shuttles electrons in the respiratory chain and they impair ATP synthesis and growth by competitive inhibition of key respiratory enzymes (Nowicka and Kruk, 2010; Szamosvári et al., 2020). The results reported here support that hypothesis and suggest that environmental bacteria that only produce naphthoquinones for aerobic metabolism are more likely to be inhibited by *B. pseudomallei* in zone of inhibition and coculture competition assays than bacteria that produce benzoquinones or benzoquinones and naphthoquinones (Figure 3 and Table 1). The predominant respiratory quinones produced by the environmental *Bacteroidota* isolates in this study are MK-6 (R12, R26, and RZ23) and MK-7 (ST4, ST5, ST6, and ST7) (Collins and Jones, 1981; Ntougias et al., 2007; Wang et al., 2021). All of these bacteria were inhibited by the products of the *B. pseudomallei hmqA-G* locus in zone of inhibition assays (Table 1). R12 and RZ23, on the other hand, were the only *Bacteroidota* isolates not inhibited in coculture competition assays (Supplementary Figure 3). It is possible that these bacteria inhibit the production or export of AQs by *B. pseudomallei*. While no environmental *Pseudomonadota* isolates were identified that were inhibited by the *B. pseudomallei hmqA-G* locus in both zone of inhibition and coculture competition assays (Table 1), only 32 strains were evaluated and it is possible that such isolates could be identified in a more exhaustive environmental survey.

Transposon mutagenesis was used to identify two distinct loci in Bp82 that were responsible for competitive fitness in coculture with environmental Gram-negative bacteria in the *Bacteroidota* phylum. Three mutations were identified in the *hmqA-G* locus and two mapped to the *scmR* regulatory gene (Figure 2). LC-MS/MS experiments confirmed that both loci are absolutely essential for the production of AQs, AQNOs, MAQs, and MAQNOs (Figure 2D and Table 2). The secondary metabolite regulator, ScmR, is a LTTR that was originally identified in *B. thailandensis* (Mao et al., 2017). The *hmqA-G* gene cluster is positively regulated by ScmR in *B. thailandensis* (Mao et al., 2017), but this study is the first to evaluate the importance of ScmR for *hmqA-G* regulation in *B. pseudomallei*. β-galactosidase assays were utilized to evaluate *hmqD-lacZ* and *scmR-lacZ* transcriptional fusions in Bp82 and the results confirmed that ScmR is a positive regulator of the *hmqA-G* locus in *B. pseudomallei* (Figure 4A). In addition, the transcription of *scmR* and *hmqA-G* are both activated by QS in *B. thailandensis* and *B. pseudomallei* (Figure 4B; Klaus et al., 2018; Le Guillouzer et al., 2020; Majerczyk C. et al., 2014; Majerczyk C. D. et al., 2014; Martinez et al., 2020). All three of *B. pseudomallei*’s AHLs, C_8_-HSL, 3OH-C_10_-HSL, or 3OH-C_8_-HSL, activated the transcription of *scmR* (Figure 5). Taken together, the results suggest that the activation of *scmR* expression by QS is followed by the activation of *hmqA-G* transcription by ScmR. An in-depth analysis of the production of Δ^2^-MNQ, MNQ, and Δ^2^-MNQNO during the growth of Bp82 and CM139 revealed some interesting differences (Figure 6). The production of all three molecules peaked at the late exponential to early stationary phase of growth in Bp82 and then steadily declined throughout the stationary phase (Figures 6B–D). The production of MAQs by *B. ambifaria* followed a similar trend when grown aerobically (Prothiwa et al., 2021). By comparison, the production of Δ^2^-MNQ and MNQ by CM139 steadily increased during the stationary phase of growth (Figures 6B, C). One possible explanation for the production differences of these compounds is that there may be a QS-activated MAQ and MAQNO degradation mechanism occurring in Bp82 that is not upregulated in CM139 during the stationary phase of growth. Alternatively, the Δ^2^-MNQ and MNQ production differences could be due to the QS-mediated levels of ScmR. The *scmR* gene in *B. thailandensis* is negatively autoregulated and it is possible that this is also true in *B. pseudomallei* (Le Guillouzer et al., 2020). If this is the case, the expression of *scmR* in Bp82 likely hits a threshold level in early stationary phase where it automatically shuts down and leads to a decrease in *hmqA-G* transcription and a decrease in Δ^2^-MNQ and MNQ production. CM139 may never reach the threshold level of *scmR* expression during the stationary phase of growth and negative autoregulation may not occur. As a result, ScmR may remain at sufficient levels to continually activate the transcription of *hmqA-G* and the production of Δ^2^-MNQ and MNQ. Finally, CM139 did not produce AQNOs or MAQNOs, including Δ^2^-MNQNO, during any stage of growth (Figure 8D and Table 2). The *hmqL* gene encodes a *N*-hydroxylating flavoprotein monooxygenase that converts AQs and MAQs into *N*-oxides (Ernst et al., 2022; Klaus et al., 2020b; Savchenko et al., 2024). This gene is not linked to the *hmqA-G* gene cluster and it may be differentially regulated by QS and ScmR. The LC-MS/MS results suggest that this gene may not be transcribed or the encoded enzyme may not be stable in the QS-deficient strain CM139. Further studies will be required to understand why this strain does not produce AQNOs or MAQNOs.

The importance of AQ derivatives for *B. pseudomallei* survival and replication within host cells was assessed in this study using 1026b and 1026b *ΔhmqD*. The results showed that while 1026b *ΔhmqD* persisted intracellularly during the 12 h experiment, it did not replicate as well as 1026b inside the murine macrophage cell line RAW264.7 (Figure 7A). Thus, the intracellular production of AQs, AQNOs, MAQs, and MAQNOs by *B. pseudomallei* provided an unexpected replication benefit within this niche. *B. thailandensis* was recently shown to produce a variety of secondary metabolites, including AQs, MAQs, MAQNOs, when grown inside RAW264.7 cells (Aiosa et al., 2022). It is tempting to speculate that these molecules also promote the replication of this facultative intracellular bacterium, but additional studies are required to address this possibility. Eukaryotic targets of the AQ derivatives include NADH:ubiquinone oxidoreductase (complex I) and ubiquinol:cytochrome c oxidoreductase (complex III) of the mitochondrial respiratory chain (Reil et al., 1997) and the pyrimidine biosynthesis enzyme dihydroorotate dehydrogenase (DHODH) (Garrett and Whalen, 2023). These enzymatic targets all interact with UQ and the AQs, AQNOs, MAQs, and MAQNOs compete with UQ binding to disrupt electron transport and pyrimidine biosynthesis (Horwitz et al., 2022; Wu and Seyedsayamdost, 2017). Fungi are also inhibited by these *Burkholderia* secondary metabolites and it is likely that the same enzymes are targeted (Kilani-Feki et al., 2012; Moon et al., 1996; Wang et al., 2020). We hypothesize that AQ derivatives are exported inside the RAW264.7 cell cytosol by intracellular *B. pseudomallei* where they competitively inhibit these enzymes and provide a replication advantage. Further studies will be required to test this hypothesis.

*B. pseudomallei* 1026b *ΔhmqD* was notably less virulent than 1026b in the murine model of melioidosis indicating that AQs, AQNOs, MAQs, and MAQNOs are important *B. pseudomallei* virulence factors (Figure 7B). This is the first time that the *B. pseudomallei hmqA-G* locus has been shown to be important for mammalian pathogenesis. Previous studies have also suggested that the products of the *hmqA-G* locus might be involved in virulence. When *B. pseudomallei* JW270 and JW270 *ΔhmqD* were assessed in the Madagascar hissing cockroach model of infection, the LD_50_ of JW270 *ΔhmqD* was found to be > 10^3^ times higher than JW270 (Chua et al., 2022) suggesting that *Burkholderia* quinolones are also important for virulence in different host species. In addition, Price et al. found that the *B. pseudomallei hmqA-G* genes were upregulated in chronically adapted cystic fibrosis (CF) patient isolates following growth in artificial CF sputum medium (Price et al., 2018). The reason for the decreased virulence of *B. pseudomallei* 1026b *ΔhmqD* may be due to the intracellular replication defect in macrophages mentioned above or it could be due to another pathogenesis-associated deficiency. The first five genes of the *B. pseudomallei hmqA-G* locus encode proteins that are homologous to the products of the *Pseudomonas aeruginosa pqsA-E* locus (Diggle et al., 2006; Vial et al., 2008). This gene cluster in *P. aeruginosa* encodes for greater than 50 distinct 2-alkyl-4-quinolones (AHQs), including 2-heptyl-3-hydroxy-4-quinolone (PQS) and 2-heptyl-4-quinolone (HHQ) (Déziel et al., 2004; Diggle et al., 2003). PQS, also known as *Pseudomonas* Quinolone Signal, is important for QS-mediated expression of virulence factors and a variety of QS-independent activities (Lin et al., 2018). Importantly, homologs of *B. pseudomallei hmqE* and *hmqG* are not present in *P. aeruginosa* and the AHQ molecules produced by this organism do not contain a methyl group at position 3 or an alkyl group with unsaturation at position 2’ (Agarwal et al., 2012; Savchenko et al., 2024; Vial et al., 2008). This could be important as different chemical substitutions on the quinolone core scaffold can result in substantial differences in biological activity (Saalim et al., 2020). Unlike the *P. aeruginosa pqsA-E* gene cluster, there is limited data on the role of the *B. pseudomallei hmqA-G* locus in QS. Diggle et al. demonstrated that a *B. pseudomallei hmqA* mutant exhibited a change in colony morphology and elevated elastase production and both phenotypes were attributed to altered AQ signaling (Diggle et al., 2006). However, *B. pseudomallei* Bp82 *ΔhmqD* did not exhibit an altered colony phenotype or elevated elastase production relative to Bp82 in this study, but it did not produce a zone of clearing on 3% skim milk agar plates (data not shown). Multiple *B. pseudomallei* peptidases and proteases are exported by the type II secretion system and are responsible for the zone of clearing on skim milk agar plates (Burtnick et al., 2014). The reason for the defect in peptidase and protease export by Bp82 *ΔhmqD* is unknown, but it could be due to altered AQ signaling. Genome-wide transcriptional profiling studies need to be conducted to better understand the potential role of *B. pseudomallei* AQs in QS-mediated gene expression before any conclusions can be made about the involvement of these molecules in cell density-mediated phenotypes. While the exact molecular mechanism of their role in virulence is currently unknown, the results presented here clearly demonstrate that the AQ, AQNO, MAQ, and MAQNO molecules produced by *B. pseudomallei* are critical for competitive fitness against environmental Gram-negative bacteria in the phylum *Bacteroidota* and for pathogenesis in the murine model of melioidosis. In addition, inhibition of *B. pseudomallei* AQ production by HmqD inhibitors like compound 4 (Figure 8) could represent a novel therapeutic countermeasure for melioidosis patients in the future.

## Data Availability

The GenBank accession numbers for the Gram-negative 16S rRNA sequences described in this study are PP920381-PP920419.

## Author disclaimer

The opinions, interpretations, conclusions, and recommendations presented are those of the authors and are not necessarily endorsed by the U.S. Army or the Defense Health Agency.

Research was conducted under an Institutional Animal Care and Use Committee (IACUC) approved protocol in compliance with the Animal Welfare Act, PHS Policy, and other Federal statutes and regulations relating to animals and experiments involving animals. The facility where this research was conducted is accredited by the Association for Assessment and Accreditation of Laboratory Animal Care International and adheres to principles stated in the *Guide for the Care and Use of Laboratory Animals*, National Research Council, 2011.

3-Methyl-2-alkylquinolones (MAQs) have been also termed HMAQs referring to their tautomeric 4-hydroxy-3-methyl-2-alkyl-4(1H)-quinolone form. To be consistent with the nomenclature of 2-alkyl-4(1H)-quinolones (AQs), we chose the abbreviation MAQs and MAQNOs for the corresponding *N*-oxides.
