# Supplementary material for: Burkholderia pseudomallei produces 2-alkylquinolone derivatives important for host virulence and competition with bacteria that employ naphthoquinones for aerobic respiration
Source: Front Microbiol. 2024 Oct 14;15:1474033. doi: 10.3389/fmicb.2024.1474033 (PMC11513363; doi:10.3389/fmicb.2024.1474033)
Supplement: Supplementary file 1 [file Data_Sheet_1.PDF]

Supplementary Figures and Tables

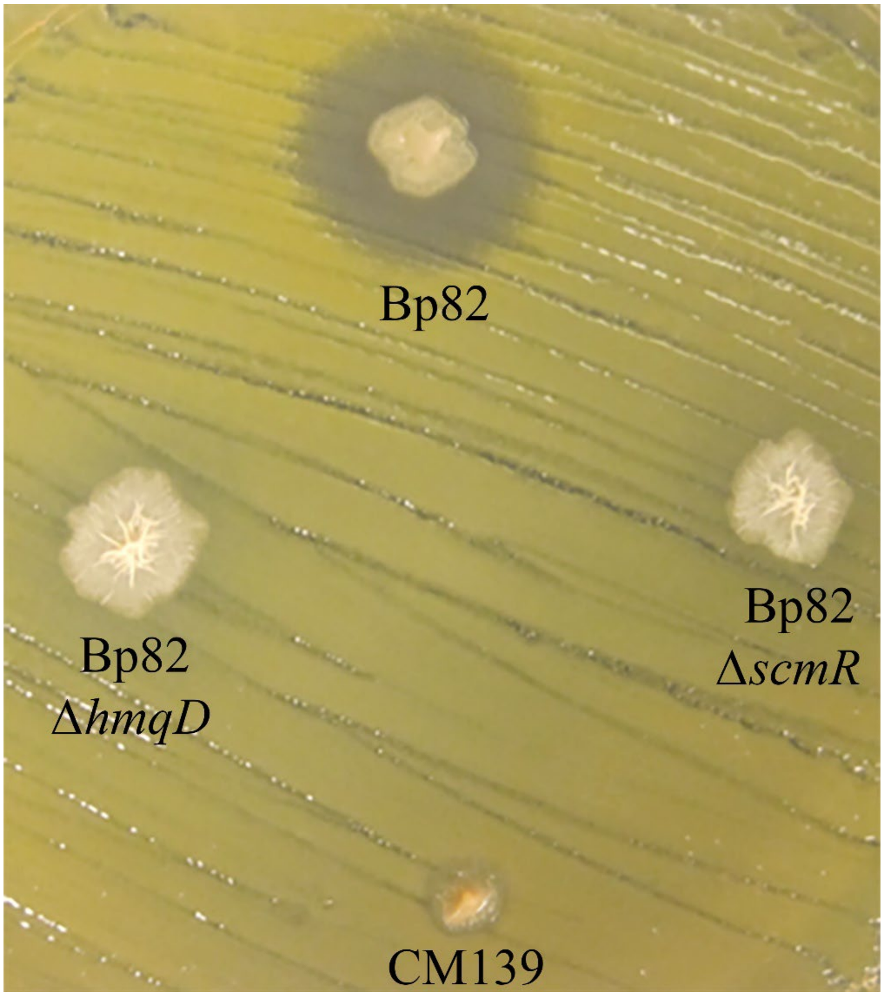

Supplementary Figure 1. Bp82 produces a zone of inhibition on a lawn of *Sphingobacterium* sp. ST4, but Bp82 Δ*hmqD* and Bp82 Δ*scmR* are unable to produce such zones. CM139 produces a thin, but noticeable, zone of clearing on a lawn of ST4. The inoculated LB agar plate was incubated for 24 h at 37˚C.

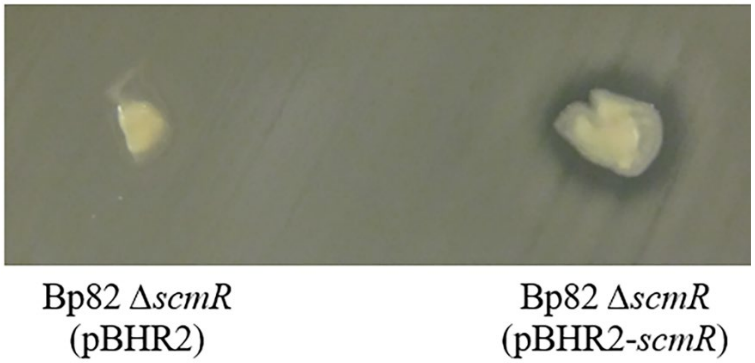

Supplementary Figure 2. Bp82 Δ*scmR* (pBHR2-*scmR*) produces a zone of inhibition on a lawn of *Sphingobacterium* sp. ST4, but Bp82 Δ*scmR* (pBHR2) does not. The inoculated LB agar plate was incubated for 24 h at 37˚C.

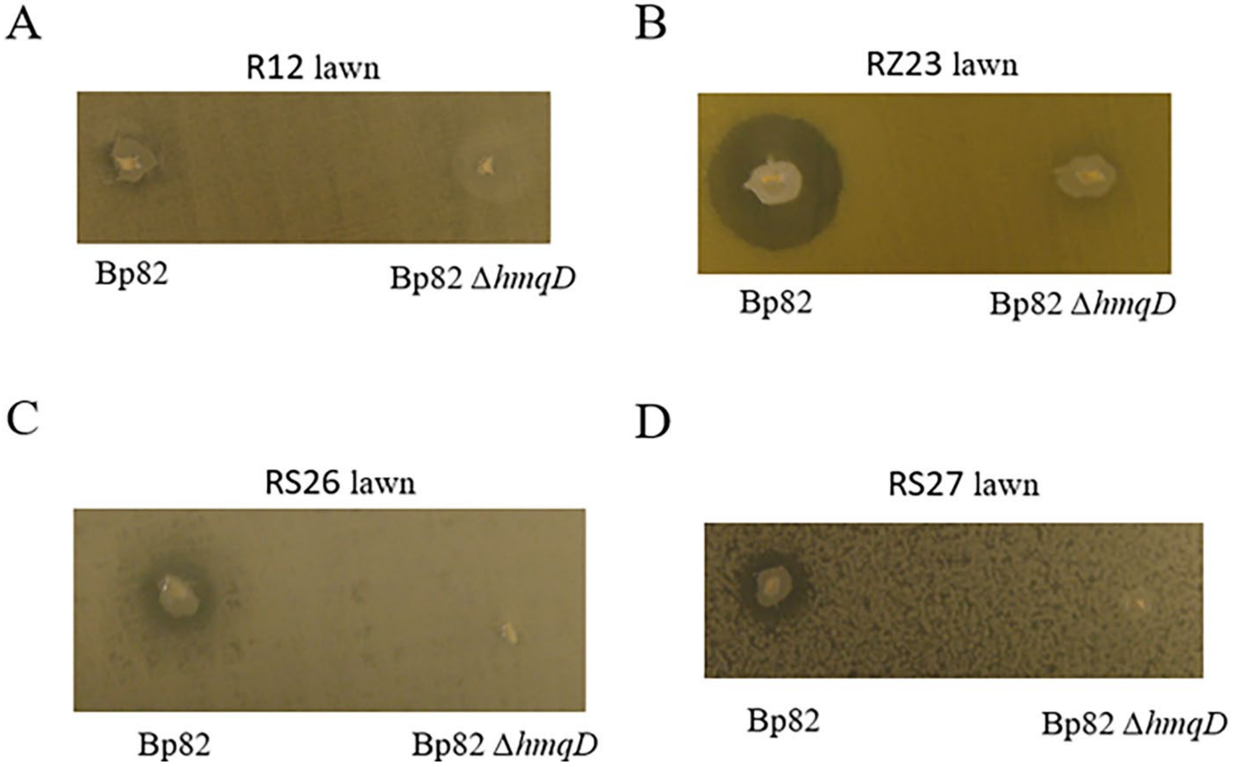

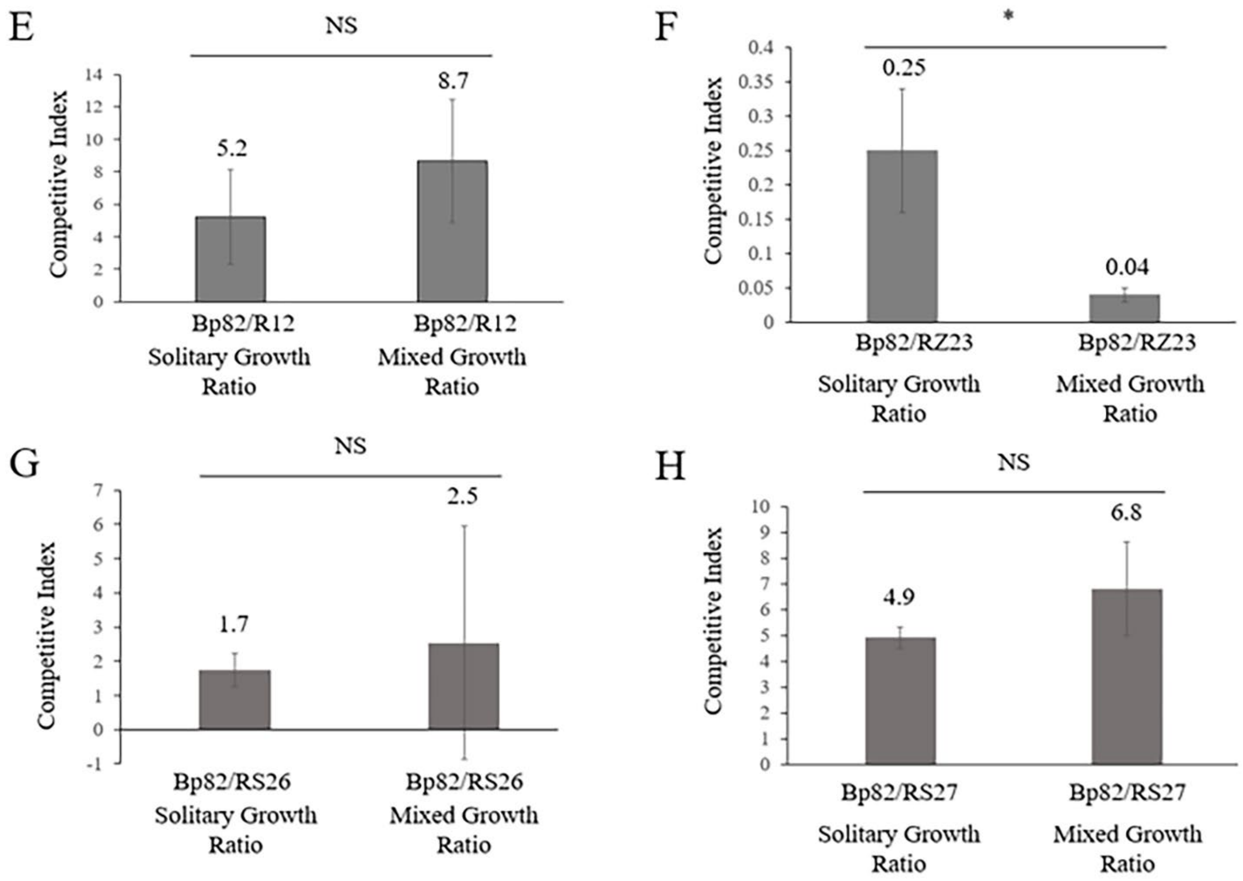

Supplementary Figure 3. R12, RZ23, RS26, and RS27 were inhibited by Bp82 in a *hmqD*-dependent manner when grown on agar lawns (A-D), but were not inhibited by Bp82 in a coculture competition assay (E-H). For the coculture assay, the fold difference between the *B. pseudomallei*/environmental isolate ratio when the bacteria were grown alone (solitary growth ratio) or in mixed culture (mixed culture ratio) was used to establish the competitive indexes. The bacteria were incubated at RT for 48 h on solid medium and the surviving competitors were quantitated. Three independent pairs of cultures were performed for each competition assay and the results were recorded as the mean ± the standard deviation. NS, not significant; *, P <0.01.

Supplementary Table 1. Strains, plasmids, and primers used in this study

Strain or plasmid	Relevant characteristics[a]	Source or Reference
E. coli		
TOP10	General cloning and blue/white screening	Life Technologies
E. cloni® 10G	General cloning and blue/white screening	Lucigen
INV110	*dam* and *dcm* deficiencies to allow restriction digestion with Dam- and Dcm-sensitive restriction enzymes	Invitrogen
S17-1	Mobilizing strain with transfer genes of RP4 integrated on chromosome; Sm^r, Tp^r	(Simon et al., 1989)
B. pseudomallei		
1026b	Isolated in Thailand from a human case of septicemic melioidosis with skin, soft tissue, and spleen involvement; Pm^r	(DeShazer et al., 1997)
Bp82	1026b *ΔpurM* derivative; adenine and thiamine auxotroph	(Propst et al., 2010)
CCM1	Bp82 derivative; *scmR*::Tn*Mod*-OKm'	This study
CCM2	Bp82 derivative; *scmR*::Tn*Mod*-OKm'	This study
CCM6	Bp82 derivative; *hmqB*::Tn*Mod*-OKm'	This study
CCM7	Bp82 derivative; *hmqF*::Tn*Mod*-OKm'	This study
CCM9	Bp82 derivative; *hmqF*::Tn*Mod*-OKm'	This study
Bp82 Δ*scmR*	Bp82 derivative harboring a 967-bp deletion mutation in *scmR* (Δ*scmR*)	This study
Bp82 *scmR-lacZ*	Bp82 containing a *scmR-lacZ* transcriptional fusion	This study
Bp82 Δ*hmqD*	Bp82 derivative harboring a 318-bp in-frame deletion mutation in *hmqD* (Δ*hmqD*)	(Mou et al., 2021)
Bp82 *hmqD-lacZ*	Bp82 containing a *hmqD-lacZ* transcriptional fusion	This study
Bp82 *hmqD-lacZ* Δ*scmR*	Bp82 *hmqD-lacZ* containing a 269-bp in-frame deletion of *scmR*	This study
CM139	Bp82 derivative; Δ*bpsI1*, Δ*bpsI2*, Δ*bpsI3*	(Majerczyk et al.,

		2014)
CM139 *hmqD-lacZ*	CM139 containing a *hmqD-lacZ* transcriptional fusion	This study
CM139 *hmqD-lacZ* Δ*scmR*	CM139 *hmqD-lacZ* containing a 269-bp in-frame deletion of *scmR*	This study
CM139 *scmR-lacZ*	CM139 containing a *scmR-lacZ* transcriptional fusion	This study
Plasmids		
pCR2.1-TOPO	3,931-bp TA vector; pMB1 *oriR*, Km^r, Ap^r	Life Technologies
pCR2.1-*lacZ*	pCR2.1-TOPO containing promoterless *lacZ* gene from *E. coli*	(Losada et al., 2018)
pCR2.1-Δ*hmqD*	pCR2.1-TOPO containing *hmqD* with a 318-bp in-frame deletion	(Mou et al., 2021)
pCR2.1-Δ*hmqD-lacZ*	pCR2.1-Δ*hmqD* digested with *Cla*I, blunted, and ligated to *lacZ* insert from pCR2.1-*lacZ* digested with *Xba*I and *Bam*HI and blunted	This study
pCR2.1-*scmR*	pCR2.1-TOPO containing PCR product generated with scmR-up and scmR-dn	This study
pCR2.1-*scmR*2	pCR2.1-TOPO containing PCR product generated with ScmR Forward and ScmR Reverse	This study
pTn*Mod*-OKm′	5,098-bp minitransposon vector; pMB1 *oriR*; RP4 *oriT*; Tn*5* *tnp*; Km^r	(Dennis and Zylstra, 1998)
pCCM1As	Plasmid obtained from CCM1 by *in vitro* cloning with *Asc*I	This study
pCCM1Sa	Plasmid obtained from CCM1 by *in vitro* cloning with *Sal*I	This study
pCCM2As	Plasmid obtained from CCM2 by *in vitro* cloning with *Asc*I	This study
pCCM2Sa	Plasmid obtained from CCM2 by *in vitro* cloning with *Sal*I	This study
pCCM6N	Plasmid obtained from CCM6 by *in vitro* cloning with *Not*I	This study
pCCM7N	Plasmid obtained from CCM7 by *in vitro*	This study

	cloning with *Not*I	
pCCM9Sa	Plasmid obtained from CCM9 by *in vitro* cloning with *Sal*I	This study
pMo130	6,127-bp suicide vector for allelic exchange in *Burkholderia*; ColE1 *oriR,* RK2 *oriT*, *xylE*, *sacB*; Km^r	(Hamad et al., 2009)
pMo130-Δ*hmqD*-*lacZ*	pMo130 digested with *Xba*I and containing *Xba*I-*Spe*I insert from pCR2.1-Δ*hmqD*-*lacZ*	This study
pMo130-*scmR*	pMo130 digested with *Nhe*I and *Xba*I and containing *Nhe*I insert from pCR2.1-*scmR*	This study
pMo130-Δ*scmR*	pMo130-*scmR* digested with *Sal*I and re-ligated without the 269-bp insert	This study
pMo130-Δ*scmR*-*lacZ*	pMo130-Δ*scmR* digested with *Sal*I, blunted, and ligated to *lacZ* insert from pCR2.1-*lacZ* digested with *Xba*I and *Bam*HI and blunted	This study
pMo130-*ΔscmR*2	pMo130 containing scmR-5'-F/-R and scmR-3'-F/-R PCR products joined by *SOE*	This study
pBHR2	6,702-bp broad-host-range plasmid; pBBR1 *oriR*, *oriT*, Km^r	(Schell et al., 2007)
pBHR2-*scmR*	pCR2.1-*scmR*2 digested with *Eco*RI and *Hind*III and cloned into corresponding sites of pBHR2	This study
Primers (5'-3')		
553F	GTGCCAGCAGCCGCGGTAA	(Weisburg et al., 1991)
1492R	ACCTTGTTACGACTT	(Lane, 1991)
M13 Forward	GTAAAACGACGGCCAG	Life Technologies
M13 Reverse	CAGGAAACAGCTATGAC	Life Technologies
KM-RT	CTTCACGAGGCAGACCTCAG	This study
TnMod-LT2	TTCCTGGTACCGTCGACATG	(Mou et al., 2021)
hmqD-up	TCGCCGCGGCGTCGTGAAGC	(Mou et al., 2021)
lacZ-dn2	TTATGCAGCAACGAGACGTC	This study
scmR-5'-F	CAGGGCCCCGCTAGCGATGATCTTCG CCATCG	This study

scmR-5'-R	CACCTCTAGACGACGGTTCATAGCTT TGCTTG	This study
scmR-3'-F	CGTCGTCTAGAGGTGCAGTGACGCCC GACTGAAGT	This study
scmR-5'-R	GAAGATCTGGCTAGCTAATCGTTCGC CGATC	This study
scmR-up	GCTAGCGATTAAATCCGGCGCAATCG	This study
scmR-dn	GCTAGCGAGATCGACGTGGTTGAAG C	This study
ScmR Forward	GAATTCGATATCATGAACCAAATCCA GACCATGCGTG	This study
ScmR Reverse	AATAATAAGCTTTCACTGGAGCCCGG TGACG	This study

[a] r, resistant; s, susceptible; Sm, streptomycin; Tp, trimethoprim; Pm, polymyxin B; Km, kanamycin; Ap, ampicillin

References

Dennis, J. J. and Zylstra, G. J. (1998). Plasposons: modular self-cloning minitransposon derivatives for rapid genetic analysis of Gram-negative bacterial genomes. *Appl. Environ. Microbiol.* 64**,** 2710-2715.

DeShazer, D., Brett, P. J., Carlyon, R. and Woods, D. E. (1997). Mutagenesis of *Burkholderia pseudomallei* with Tn*5*-OT182: Isolation of motility mutants and molecular characterization of the flagellin structural gene. *J. Bacteriol.* 179**,** 2116-2125.

Hamad, M. A., Zajdowicz, S. L., Holmes, R. K. and Voskuil, M. I. (2009). An allelic exchange system for compliant genetic manipulation of the select agents *Burkholderia pseudomallei* and *Burkholderia mallei*. *Gene*. 430**,** 123-131.

Lane, D. J. (1991). "16S/23S rRNA sequencing" in *Nucleic acid techniques in bacterial systematics*, ed. Stackebrandt, E. & Goodfellow, M., (New York: John Wiley & Sons)**,** 115-175.

Losada, L., Shea, A. A. and DeShazer, D. (2018). A MarR family transcriptional regulator and subinhibitory antibiotics regulate type VI secretion gene clusters in *Burkholderia pseudomallei*. *Microbiology*. 164**,** 1196-1211.

Majerczyk, C. D., Brittnacher, M. J., Jacobs, M. A., Armour, C. D., Radey, M. C., Bunt, R., et al. (2014). Cross-species comparison of the *Burkholderia pseudomallei*, *Burkholderia thailandensis*, and *Burkholderia mallei* quorum-sensing regulons. *J. Bacteriol.* 196**,** 3862-3871.

Mou, S., Jenkins, C. C., Okaro, U., Dhummakupt, E. S., Mach, P. M. and DeShazer, D. (2021). The *Burkholderia pseudomallei hmqA-G* locus mediates competitive fitness against environmental gram-positive bacteria. *Microbiol. Spectr*. 9**,** e0010221.

Propst, K. L., Mima, T., Choi, K. H., Dow, S. W. and Schweizer, H. P. (2010). A *Burkholderia pseudomallei* Δ*purM* mutant is avirulent in immunocompetent and immunodeficient animals: candidate strain for exclusion from select-agent lists. *Infect. Immun.* 78**,** 3136-3143.

Schell, M. A., Ulrich, R. L., Ribot, W. J., Brueggemann, E. E., Hines, H. B., Chen, D., et al. (2007). Type VI secretion is a major virulence determinant in *Burkholderia mallei*. *Mol. Microbiol.* 64**,** 1466-1485.

Simon, R., Quandt, J. and Klipp, W. (1989). New derivatives of transposon Tn*5* suitable for mobilization of replicons, generation of operon fusions and induction of genes in gram-negative bacteria. *Gene*. 80**,** 161-169.

Weisburg, W. G., Barns, S. M., Pelletier, D. A. and Lane, D. J. (1991). 16S ribosomal DNA amplification for phylogenetic study. *J. Bacteriol.* 173**,** 697-703.
